# Photocatalytic Biohybrid Vesicles

**DOI:** 10.1021/acs.chemrev.5c00808

**Published:** 2026-01-14

**Authors:** Julea N. Butt, Lars J. C. Jeuken

**Affiliations:** † School of Biological Sciences, 6106 University of East Anglia, Norwich Research Park, Norwich NR4 7TJ, United Kingdom; ‡ Leiden Institute of Chemistry, Leiden University, PO Box 9502, 2300 RA Leiden, The Netherlands

## Abstract

Semiartificial photosynthesis presents an attractive route to overcome limitations of natural photosynthesis for sustainable chemicals production. Synthetic materials are combined with biological molecules, forming biohybrid systems, that provide unique opportunities to innovate new solar-to-chemical pathways. There are further advantages if the biohybrids confine specific processes to different spatial locations. Such behavior is a defining feature of natural photosynthesis and it is mimicked in the photocatalytic biohybrid vesicles discussed in this Review. A nonleaky membrane comprised of amphiphilic molecules defines the wall of the reactor vesicle. Light-driven directional transfer of electrons and/or ions across the vesicle membrane generates an (electro)­chemical gradient, a form of energy storage, that is subsequently harnessed for chemical synthesis. In such systems, nonproductive backreactions are avoided, reactants can be concentrated to favor their conversion, and reaction intermediates can be channeled through the desired pathway. This Review introduces natural photosynthesis and vesicles as biohybrid reaction containers. Different approaches to achieving light-driven charge transfer across vesicle membranes are reviewed, and state-of-the-art strategies for delivering light-driven chemical production are systematically summarized for this interdisciplinary field. Finally, key scientific problems and bottlenecks to the development of photocatalytic biohybrid vesicles are defined to provide insights for driving forward future research.

## Graphical abstract

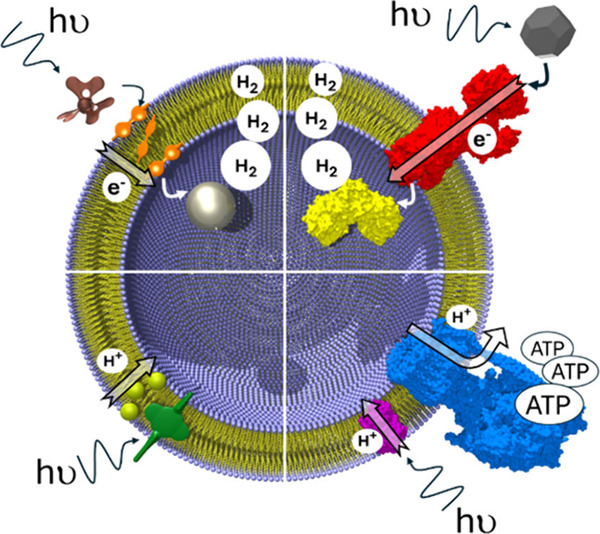

## 1 Introduction

In the drive for sustainability, contemporary processes for chemicals manufacturing present two significant challenges. First, most products are derived from crude oil or natural gas. Second, production of these petrochemicals is very energy intensive. As a consequence, converting renewable feedstocks to useful chemicals by direct harnessing of the energy in sunlight, our most abundant and sustainable energy source, presents an attractive route to defossilizing the chemicals industry.^1–3^ Natural photosynthesis provides an inspiring blueprint for such solar to chemicals conversion since, following light-absorption and charge separation, water and carbon dioxide (CO_2_) are converted to oxygen (O_2_) and complex multicarbon molecules, Figure 1. However, natural photosynthesis suffers disadvantages for commercial chemicals production.^4^ Most of the chemicals in biomass, being carbohydrates, have little commercial value. Extraction of valuable chemicals from the complex mixtures in biomass is both costly and time-consuming. In addition, for land-based photosynthesis the potential competition with crop production is such that there is little practical utility to meet global chemical demands. Thus, while the advantageous catalytic capabilities of biology have delivered resilient production of complex molecules they are unlikely to produce industrial chemicals on a scale required to meet net zero targets.

**Figure 1:**
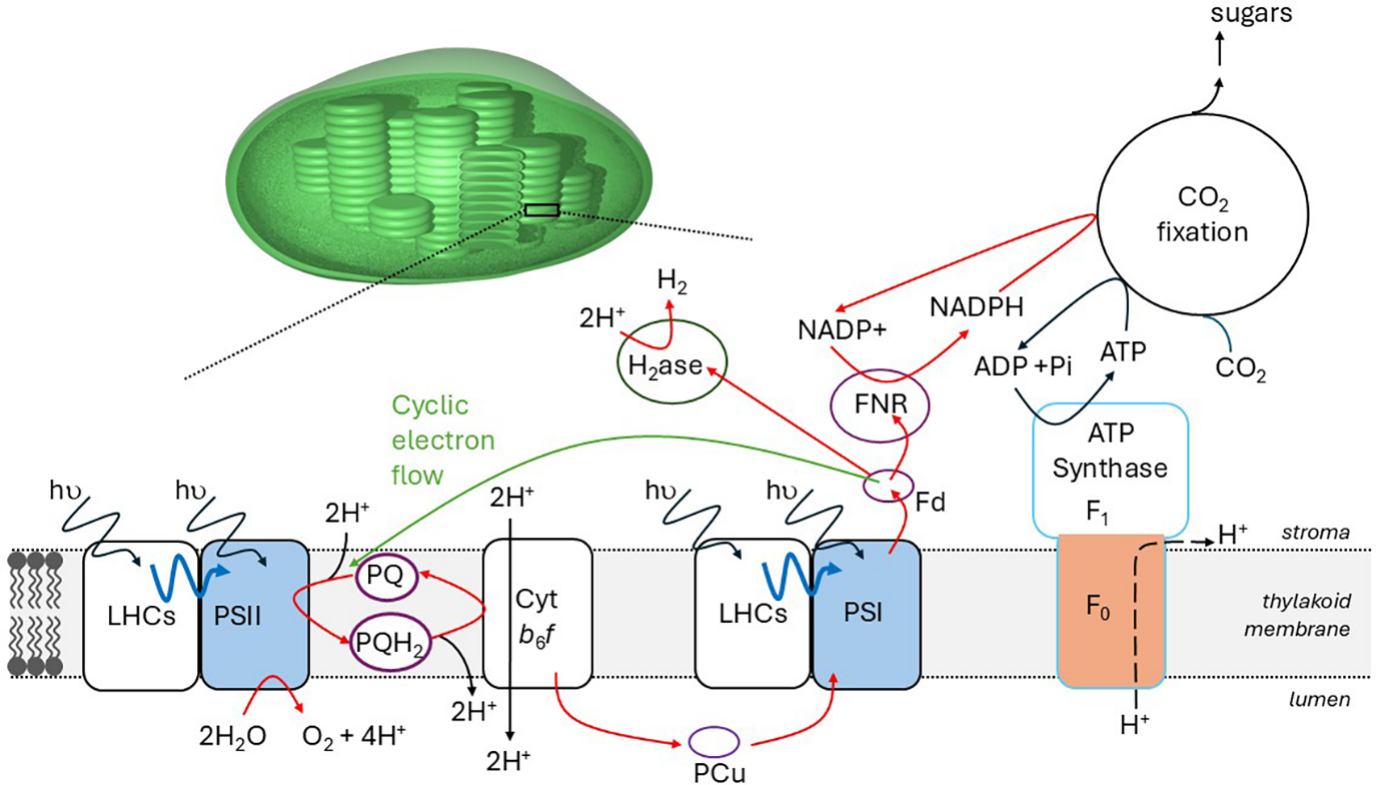
A schematic representation of the photosynthetic system in green plant chloroplasts. ADP = adenosine 5′-diphosphate; ATP = adenosine 5′-triphosphate; Cyt *b*_6_ *f* = cytochrome *b*_6_ *f*; H_2_ase = hydrogenase; Fd = ferredoxin; FNR = ferredoxin-NADP^+^ oxidoreductase; LHCs = light-harvesting complexes; NADP^+^/NADPH = nicotinamide adenine dinucleotide phosphate; PCu = plastocyanin; Pi = phosphate; PSI & PSII = photosystem I & II, PQ = plastoquinone.

Semiartificial photosynthesis presents an attractive way to overcome the limitations of natural photosynthesis for chemicals production.^5–11^ Here synthetic materials are combined with biological molecules to form biohybrid systems that present unique opportunities to innovate new solar-to-chemical pathways. Complementary tasks are delegated to components that must be effectively interfaced to achieve an optimal outcome. The aim is not to reproduce the precise reactions of natural photosynthesis. Instead, economically useful molecules should be synthesized in processes where catalysis is a result of light-harvesting. Enzymes may be employed as naturally evolved catalysts performing complex chemistry with high selectivity and efficiency. Synthetic materials may provide robust broadband absorbers of high intensity light. There are further advantages to be gained if biohybrids confine specific processes to different spatial locations.^12,13^ This behavior is a defining feature of natural photosynthesis, Figure 1, and it is mimicked in the light-driven vesicular biohybrid reaction containers that form the focus of this review.

Where light-driven charge separation is followed by catalytic oxidation and reduction chemistry, employing a vesicle membrane to separate the aqueous phase for reductive chemistry from that for oxidative chemistry allows both processes in principle to occur in their optimal condition. Nonproductive back reactions are avoided, reactants can be concentrated to favor their conversion, and reaction intermediates can be channeled through the desired pathway. Further, charge transfer across the vesicular membrane creates (electro)­chemical gradients, a form of energy storage utilized downstream for chemical synthesis. By generating an (electro-)­chemical gradient across the vesicle membrane, biohybrid approaches mimic an important feature of natural photosynthesis that can be exploited for solar-to-chemicals conversion and that is not accessible when the reactions are not compartmentalized. In these ways the light-driven chemistry in vesicular biohybrid containers is a vectorial process that promises high solar conversion efficiency when compared to homogeneous approaches.

For these advantages to be realized a nonleaky membrane comprised of amphiphilic molecules defines the wall of the reactor (equivalent to the lipid bilayer of chloroplasts, Figure 1). Directional charge-transfer between the catalysts and through the hydrophobic membrane interior is then enabled by a membrane soluble carrier. That carrier may diffuse across the membrane in which case it is typically referred to as a relay, shuttle or mediator (equivalent to PQ in chloroplasts, Figure 1). Alternatively, the carrier may span the membrane to provide a conduit for charge transfer between the aqueous phases (equivalent to the electron transfer properties of PSI, Figure 1). The final component needed for a light-driven biohybrid vesicle is a light-harvesting molecule that converts solar energy to chemical energy (equivalent to PSII and PSI, Figure 1). That material absorbs light to form an excited state, which drives chemical reactions and/or forms an (electro)­chemical gradient across the vesicle membrane, having an ion concentration gradient and transmembrane potential (ΔΨ) component. In the case of proton transport (for example performed by F_0_ of ATP synthase, Figure 1), this ion gradient is a pH gradient (ΔpH), which together with ΔΨ forms a proton-motive force (*pmf*) that drives a plethora of chemical reactions in biology.

Each component in a light-driven biohybrid vesicle (the membrane, the light-harvesting species, the membrane soluble charge carrier and the catalysts) can be provided by synthetic and natural materials, and in various combinations, as we illustrate in Sections 2, 3 and 4. However, we first contextualize these bioinspired developments with an overview of natural photosynthesis, followed by an introduction to the different types of amphiphiles that form membranes and the functions of these membranes in light-driven photocatalysis. We note here that light-driven vesicular reactors are of great interest beyond the realm of solar-to-chemicals conversion. They provide opportunities to gain fundamental insight into the properties of purified biological molecules in a seminatural environment.^14–16^ They provide the basis for energy transfer when developing artificial (synthetic) cells and organelles.^13,17–20^ Thus, we have drawn on studies from across these usually quite distinct areas of research to inform the content of this Review. Section 5 draws conclusions from the current-state-of-the-art and considers bottlenecks and opportunities in developing photocatalytic biohybrid vesicles.

### 1.1 Natural Photosynthesis in Green Plants

In green plants, photosynthesis occurs inside chloroplasts through a complex series of enzyme catalyzed reactions, Figure 1. Those reactions are conveniently considered to be either light-reactions or dark-reactions which operate sequentially in spatially distinct parts of the chloroplast. During the light-reactions, sunlight is absorbed and spatially separated redox reactions are coupled by transmembrane electron transfer to drive the endergonic cellular syntheses of ATP (adenosine triphosphate) and NADPH (dihydronicotinamide adenine dinucleotide phosphate). During the dark-reactions, ATP and NADPH drive the conversion of CO_2_ into multicarbon compounds.

The main components for the light-reactions are four protein complexes spanning the thylakoid membrane, membrane confined redox-active plastoquinone (PQ) and the water-soluble electron carriers plastocyanin (PCu) and ferredoxin (Fd). These molecules act together to generate a light-powered electron flux from the high-potential couple (H_2_O/O_2_ *E*_m_ = +0.80 V, all reduction potentials are versus standard hydrogen electrode (SHE), pH 7) in the lumen to the lower-potential couple (NADPH/NADP^+^ *E*_m_ = −0.32 V) on the opposite, stromal, side of the membrane. This process is associated with simultaneous formation of a transmembrane proton gradient that drives the ATP formation through the phosphorylation of ADP molecules. In the terminology that we introduced in the previous section to describe transmembrane charge carriers, PQ/PQH_2_ is a membrane confined diffusing shuttle/mediator/relay. Photosystem I (PSI) and ATP synthase provide membrane spanning conduits for selective transfer of electrons and protons, respectively.

Looking in more detail at the underlying processes, light absorption by the redox-active P680 chlorophyll molecules of photosystem II (PSII) is followed by transfer of a photoenergized electron to PQ. Water oxidation (water splitting) in the vicinity of the lumen provides an electron to the photo-oxidized P680 pair. Subsequent light-absorption and electron transfer accompanied by proton uptake from the stroma produces PQH_2_ (plastoquinol). The cytochrome *b*_6_ *f* complex oxidizes PQH_2_ with protons released to the lumen. Subsequent reduction of PCu by the cytochrome *b*_6_ *f* complex is accompanied by additional proton transfer from the stroma to the lumen via the Q-cycle. PCu is then oxidized by PSI after the latter has absorbed light and transferred a photoenergized electron to Fd. In turn, Fd passes electrons to the enzyme FNR (ferredoxin-NADP^+^ oxidoreductase) for NADPH production. The pH gradient accumulated during this electron transfer pathway is the *pmf* driving the stromal synthesis of ATP molecules as encapsulated in chemiosmotic theory.

When considering biohybrid approaches to solar conversion, it is also of note that the absorption of sunlight in natural photosynthesis is facilitated by pigment–protein complexes known as light-harvesting, or antenna, proteins that are embedded in the thylakoid membrane. These pigments, primarily chlorophyll and carotenoids in the light-harvesting complexes (LHCs), absorb light energy in the red/blue and blue/green regions of the visible spectrum, respectively. That energy is then transferred to the redox-active chlorophyll molecules of PSI and PSII (Figure 1).

The dark-reactions are a series of enzyme catalyzed reactions performing CO_2_-fixation. Termed the Calvin cycle (or Calvin-Benson-Bassham cycle), these reactions occur in the stroma and harness ATP and NADPH formed in the light-reactions.

### 1.2 Vesicles as Biohybrid Reaction Containers

In vesicle-based, light-driven biohybrid systems, different classes of membranes have been used, which will be briefly discussed here. Within the field of artificial (synthetic) cells and organelles, these different types of membranes have been extensively reviewed, and we refer to several reviews for more in-depth discussions.^21–23^ Vesicle types are named after their membrane material, which are classified as lipids (liposomes, capsosomes and vesosomes); amphiphilic block copolymers (polymersomes); mixtures of lipids and polymers (hybrid vesicles); colloid particles (colloidosomes); proteins (proteinosomes); and, less commonly, inorganics (inorganic chemical cells, iCHELLs).^21–24^ Membrane-less containers can also be prepared by liquid–liquid phase separation, such as in coacervates or water droplets in organic solvent.^22^ In the field of vesicle-based photochemistry, the focus of this review, liposomes, polymersomes, and hybrid vesicles are the most commonly used, Figure 2.

**Figure 2:**
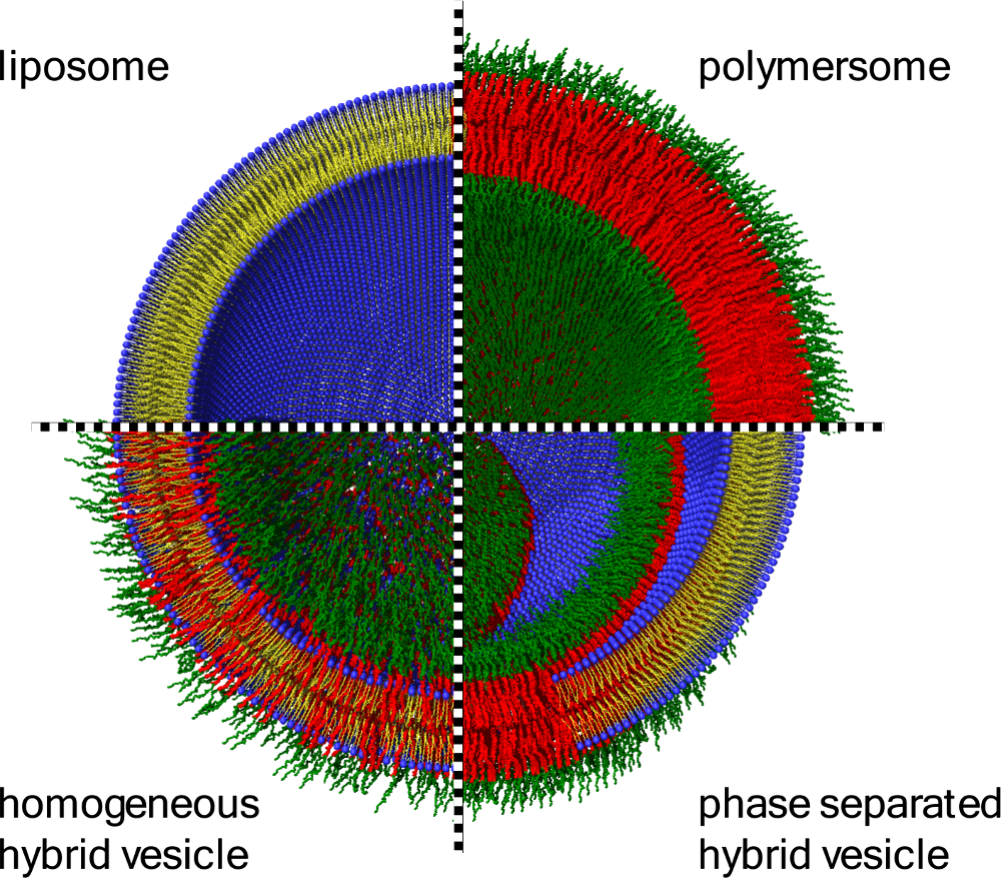
Schematic representation of three different types of vesicles, as indicated. Hybrid vesicles consisting of lipid–polymer mixtures can either form homogeneous or phase separated membranes.

Lipid vesicles, also known as liposomes, are prepared from natural or synthetic lipids. For the purpose of this review, ‘lipids’ are defined as any amphiphilic low molecular weight organic compounds that act as structural components of vesicle membranes and have a hydrophilic ‘headgroup’ and a hydrophobic ‘tail’. Besides the above-mentioned artificial (synthetic) cells, organelles and photosynthetic vesicles, liposomes have seen extensive development as drug delivery systems.^25,26^ Depending on the application, liposomes are formed with a variety of methods, most commonly but not exclusively by rehydration of dry lipid films in aqueous solution followed by mechanical treatments such as extrusion or sonication to form unilamellar vesicles. More advanced methods such as microfluidics enable better control of liposome size, lumen content and/or lipid distribution across lipid leaflets.

The large variety of natural and synthetic lipids, many commercially available, means the physicochemical properties, such as surface charge, membrane thickness, permeability, membrane fluidity and phase behavior, can be tailored. The headgroups of natural lipids are either neutral, zwitterionic or negatively charged, but synthetic lipids are used to introduce positive charges.^26^ Besides charge, a range of physicochemical and biophysical properties of liposomes are tailored with synthetic lipids,^27^ for instance lipids with poly­(ethylene glycol) (PEG) headgroups reduce biomacromolecular interactions, and lipids with chemically reactive headgroups facilitate chemical conjugations. Altering the fatty acid chain length of glycero­(phospho)­lipids, and their saturation, controls membrane permeability and fluidity, as does the inclusion of sterols, typically cholesterol. Further, many membrane proteins are functionally modulated by lipid interactions, although our understanding is still limited.^28^ Hence, lipid composition affects functionality of liposome-based biohybrid systems that include membrane proteins. Although optimization of the lipid composition of liposomal drug delivery is an intensive research field,^25,26^ few studies have systematically optimized the lipid composition of liposomes for semiartificial photosynthesis.^29,30^

Polymersomes are vesicles prepared from amphiphilic polymers, either diblock, triblock or graft copolymers. Besides semiartificial photosynthesis and biotechnology, they have potential applications in drug delivery, including programmed and targeted drug delivery, theragnostics, motion (nanomotors), stimulated response, biocatalysis and artificial cells.^16,31,32^ Diblock copolymers form bilayers, with the hydrophobic blocks forming the core of the membrane and the hydrophilic blocks on either side of the membrane, similar to lipid bilayer membranes. Triblock polymersomes are typically formed from polymers with two hydrophilic blocks either end of the polymer chain and a hydrophobic block in the middle. Triblock copolymers either span the membrane, with the hydrophilic blocks either side of the membrane, or arrange such that the hydrophilic blocks are positioned on the same side. A wide variety of copolymer compositions are known to form vesicles, although the hydrophilic block is most commonly a PEG, which in the field of polymersomes is also referred to as poly­(ethyl oxide) (PEO). Many different chemistries constitute the hydrophobic blocks, for instance poly­(1,2-butadiene), poly­(dimethylsiloxane) or poly­(cholesterol methacrylate).^31,32^

As with lipids, the chemical composition and mass of the copolymer determines the physicochemical properties of the polymersomes. The (volume) ratio between the hydrophilic and hydrophobic blocks is paramount to their ability to form vesicles.^33,34^ Higher molecular weight polymers create thicker membranes, and decrease permeability.^32^ Care must be taken with biocompatibility as many membrane proteins are not functionally active in many of the polymersomes. Polymer membranes that are significantly thicker than natural lipid membranes are thought to be less amenable to functional reconstitution of membrane proteins, although a full understanding of biocompatibility of the different copolymers is lacking.

Due to the chemical distinctiveness between lipids and copolymers, liposomes and polymersomes have very different physicochemical properties.^16^ As the variability in chemistry of both lipids and copolymers is vast, properties of, and differences between both classes of vesicles cannot be generalized. Polymersomes are often reported to be more robust than liposomes, both mechanically and chemically, but are thought to be less biocompatible, especially toward membrane proteins. In hybrid vesicles, copolymers and lipids are mixed with a view to combine the beneficial properties of both. We refer to a review by Brodszkij and Städler^31^ for an analysis of the properties of these hybrid lipid–polymer systems. The parameter space for hybrid formulations is extensive as both the lipid and copolymer chemical composition can be altered, as well as the molecular ratio between polymer and lipid. We refer to Table 1 in Brodszkij and Städler^31^ for a general description on how this affects the properties of the hybrid vesicles. Phase separation between the lipids and polymers can occur, Figure 2, and depends on the molar ratio between polymer and lipid, the environmental conditions and the type of polymers and lipids.^35–39^ Many hybrid vesicles are colloidally more stable than their liposome counterparts,^31^ while a handful of membrane proteins, not functional in polymersomes, have successfully been reconstituted in hybrid vesicles.^40–43^ We note that in some cases native membrane patches or extracts have been used to functionalize polymersomes (e.g.,^44^). In these cases lipids from the membrane patches are coinserted in the vesicle, and the resulting system could thus be seen to have a hybrid vesicle character.

**Table 1:** Early Studies on Transmembrane Charge Transfer in Liposome Systems^a^

	Donor	Membrane Mediator	Acceptor	Comments	Ref	Year
Quinones	AA	BQ, Fc	Fe(CN)_6_^3–^		^56^	1970
	NADH	UQ	O_2_		^62^	1974
	MV	MK, UQ	Various		^63^	1976
	DT	BQ, PQ, UQ, TMBQ	Fe(CN)_6_^3–^		^64^	1977
	AA					
	DT	PQ, BQ, UQ, phylloubiquinol, TMBQ, DBMIB, and others	Fe(CN)_6_^3–^		^65^,^66^	1979
	DT	MK, HQNO	Fe(CN)_6_^3–^		^67^	1993
	AA	MitoQ	Fe(CN)_6_^3–^		^68^	2016
	d-Fructose	TCNQ	Fe(CN)_6_^3–^	d-Fructose oxidation catalyzed by FDH	^69^	2016
	NADH	TCNQ	Fe(CN)_6_^3–^		^70^,^71^	2018, 2021
Mn-porphyrin	AA	Mn-hematoporphyrin	NaOCl		^72^	1976
	ITS	Quinone-linked Mn-porphyrin	Fe(CN)_6_^3–^		^73^	1988
	ITS	Phospholipid-linked Mn-porphyrin	Fe(CN)_6_^3–^		^74^	1998
Other	I^–^, DT	Polyiodine	I_2_	conduction measured in a BLM system	^75^	1970
	DT	Nickel bis(stilbenedithiolate)	Fe(CN)_6_^3–^	dicyclohexyl-18-crown-6 was used for alkali transport	^57^	1979
	H_2_	Methylene blue, 10-methyl-5-deazaisoalloxazine-3-propanesulfonic acid	Fe^3+^	Pt NP catalyzes HER	^76^	1983
	DT	(Aggregated) cytochrome *c*_3_, C_4_V	Fe(CN)_6_^3–^	Pt NP catalyzes HER	^77–79^	1981, 1982, 1984
	H_2_					
	DT	Various viologens (C_*n*_V, *n* = 1, 2, 4, 6, 8, 10, 12, 14 and 18)	Fe(CN)_6_^3–^		^60^	1984
			FMN			
	DT	MV	MV		^59^	1988
	DT	Flavolipids	Fe(CN)_6_^3–^		^80^	1989

a For abbreviations and formulas, see Figure 4. AA = ascorbic acid; BLM = black lipid membrane; DBMIB = 2,5-dibromo-3-methyl-6-isopropylbenzoquinone; DT = dithionite; Fc = ferrocene; FDH = fructose dehydrogenase; FMN = Flavin mononucleotide; HER = hydrogen evolution reaction; ITS = indigotetrasulfonic acid; NADH = nicotinamide adenine dinucleotide; HQNO = 2-heptyl-4-hydroxyquinoline N-oxide; MitoQ = Triphenylphosphonium ubi- and plastoquinones; NP = nanoparticle; TMBQ = trimethylbenzoquinone.

As mentioned, the properties of vesicles (liposomes, hybrid vesicles and polymersomes) are dependent on the type of amphiphile, or mixture of amphiphiles, and the environmental parameters such as solvent and temperature. Hence, a quantitative comparison between liposomes, hybrid vesicles and polymersomes is not possible. Still, to provide an overview of key properties of these vesicle systems, and following the approach of Brodszkij and Städler,^31^ some qualitative indicators can be given. The lateral mobility of lipids and/or polymers is lower in hybrid vesicles and polymersomes than in liposomes. Fluidity, as for instance probed by the fluorescent properties of the dye Laurdan, is often similar or lower in hybrid vesicles than liposomes. The viscoelastic properties, which represent vesicle robustness under mechanical stress, are highly dependent on the polymer studied, and parameters like the stretching/compression modulus (*K*_a_) of hybrid vesicles can be either higher or lower than liposomes. The viscoelastic properties might also depend on phase separation within hybrid vesicles. Importantly, and as already mentioned, in almost all studies the stability, i.e., shelf life, of hybrid vesicles and polymersomes are vastly superior to those of liposomes. Finally, and on first impression possibly counterintuitively, the permeability, often measured by monitoring the release of fluorescent dye, is often higher in polymersomes and hybrid vesicles than in liposomes.

### 1.3 Functionalizing Vesicles for Compartmentalized Light-Driven Chemical Synthesis

This article provides a comprehensive review of *compartmentalized*, light-driven chemical synthesis in biohybrid vesicles. In these systems vectorial charge transport takes a key role, either in compartmentalizing the photochemical reactions or by storing energy as (electro)­chemical gradients. The different strategies for light-driven vectorial transport are summarized in Figure 3 and discussed in Sections 2 and 3. Section 2 considers systems with membrane soluble diffusible redox mediators. The focus of Section 3 is on systems that employ a membrane spanning conduit for charge transfer. Section 4 extends the discussion to consider vesicular approaches to light-driven ATP production and carbon-capture, for example, through carbon-fixation. In Figure 3 we have chosen to distinguish biohybrid components of different function by using differently colored shapes. The formats were chosen to highlight analogy to the components of natural photosynthesis that we presented in Figure 1 and they are applied throughout subsequent figures to emphasize similarities and differences of the systems discussed.

**Figure 3:**
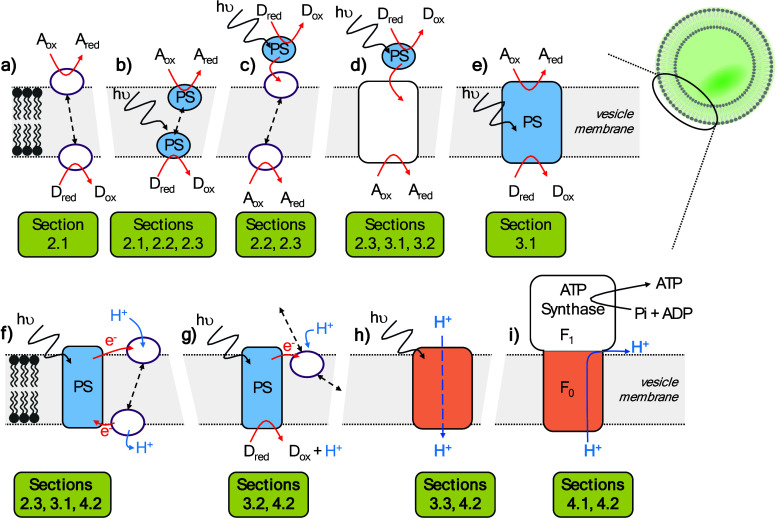
Schematic representation of the biohybrid vesicle systems that are reviewed, with the relevant sections indicated. The components are colored to show functional similarity with the components of natural photosynthesis as illustrated in Figure 1. White circles with purple borders = mediators; Blue circles = photosensitizers (PS); White squares = conduits; Blue squares = photosensitizers that transverse the membrane; Orange squares = proton-translocating conduits. A_(ox/red)_ = acceptor of electrons in oxidized and reduced states. ADP = adenosine 5′-diphosphate; ATP = adenosine 5′-triphosphate; D_(ox/red)_ = donor of electrons in oxidized and reduced states; Pi = phosphate.

Before we commence to the next section, we note that in artificial photosynthesis, vesicles and membranes fulfill several roles other than compartmentalization, and these will be briefly summarized here.^45^ First, by coupling photosensitizers and catalysts to the fluid membranes, their reaction space is confined, increasing their effective concentrations. Higher concentrations of photosensitizers and catalysts enhance their reactivity toward each other and thereby the efficiency of charge separation.^46–49^ When photocatalytic systems include unstable intermediates, enhanced charge transfer has also been shown to improve stability, thus further improving photocatalysis.^50^ Hansen et al. showed that by reducing the temperature of a lipid membrane to below the transition temperature, phase separation further enhanced local concentrations of photosensitizer and catalysts.^51^ Second, vesicles are used to help solubilize inorganic photosensitizers and catalysts. Takizawa et al. used vesicles to not only improve the solubility of poorly soluble catalysts and photosensitizers, but also to incorporate an antenna-like compound for increased light absorption.^52^ By ion-pairing an anionic Ir­(III) complex with high light absorptivity (the ‘antenna’), to a cationic Ir­(III) photosensitizer on the vesicle surface, energy transfer from the antenna to the photosensitizer was enhanced. Third, vesicles can tailor interactions with water-soluble reactants. For example, Limburg et al. showed that vesicles can control interactions between photosensitizers and reactants. Charge at the vesicles’ surface electrostatically repelled water-soluble electron acceptors from the vesicle-bound photosensitizer, thereby modifying the oxidative quenching mechanisms and reducing charge recombination.^29,30^

## 2 Photoinduced Transmembrane Charge-Transfer Using Mediators

### 2.1 Early Studies on Transmembrane Charge-Transfer Mediators

Following the chemiosmotic theory formulated by Mitchell in the early sixties,^53^ and the discovery of lipid vesicles (liposomes) in the same decade,^54,55^ transmembrane electron transfer in vesicle systems was explored in the seventies. One of the first reports of vesicle-compartmentalized redox chemistry showed reduction of encapsulated ferricyanide by extravesicular ascorbic acid. As neither ascorbic acid nor ferricyanide crosses the vesicle membrane, benzoquinone was used to ‘mediate’ electron (and proton) transfer, Figure 3a.^56^ In these and other early studies, a variety of charge mediators were identified, often with ferricyanide as an electron acceptor in the lumen of the vesicle, and either ascorbic acid or sodium dithionite as extravesicular electron donor, Table 1. For instance, Grimaldi and Lehn transported electrons with a nickel bis­(stilbenedithiolate) mediator to create a membrane potential, and then converted this potential to a concentration gradient of alkali-metals using crown ether ionophores.^57^

The most reported electron mediators are quinones and porphyrins, but a variety of other mediators have also been studied, Table 1, Figure 4, of which viologens require a special mention. In the oxidized form, viologens carry a 2+ charge and are membrane impermeable. In their singly reduced form, viologen radicals carry a 1+ charge and were expected to be membrane impermeable. However, studies have shown that the monovalent cation radical of viologens are in fact membrane permeable,^58–60^ possibly via disproportionation of two radicals.^61^ Tabushi and Kugimiya systematically changed the hydrophobicity of viologens by adding various-length alkyl chains, Figure 4.^60^ For C1–C4 chain lengths, transmembrane electron transfer rates were found to be rate limited by the phase transfer of the reduced viologen cation radical from the aqueous phase into the membrane. Oppositely, transmembrane electron transfer by longer chain viologen cation radicals (C4–C18) was rate limited by phase transfer from the membrane into the aqueous phase.^60^

**Figure 4:**
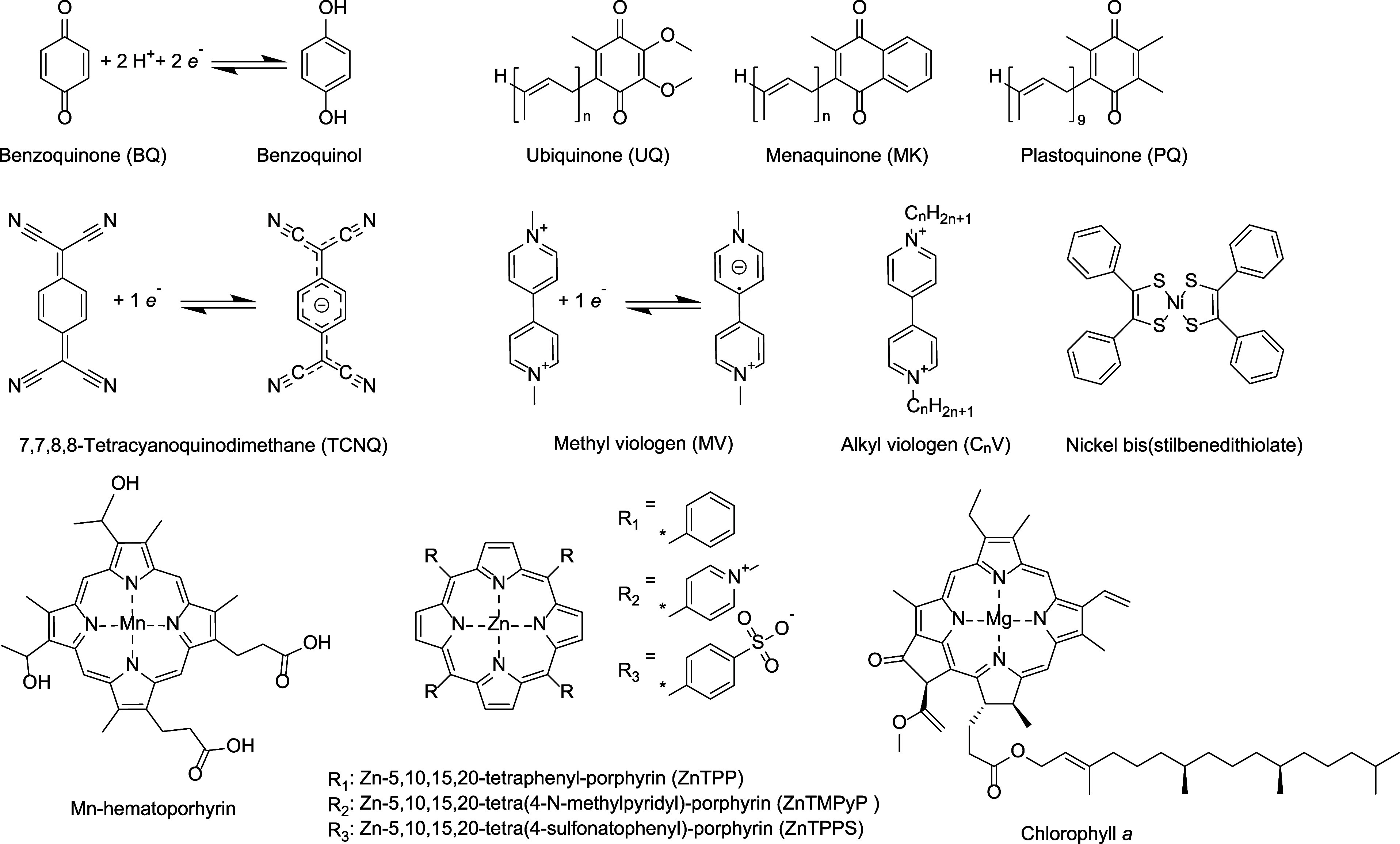
Chemical structures of mediators for transmembrane electron transport (top and middle rows) and photosensitizers (bottom row). Abbreviations used in the text are indicated.

The effect of hydrophobicity has also been studied for quinones, by variation of the hydrophobic isoprenoid side chain length, Figure 4.^64–66^ Many early experiments used natural quinones such as ubiquinones, menaquinones or plastoquinone,^62–66^ which are fully localized to the lipid membrane due to a long hydrophobic isoprenoid side chain.^81^ This restricts oxidation and reduction of the mediator to the lipid membrane-water interface, Figure 3a. Quinone analogues with shorter or no isoprenoid chain, including the earlier mentioned benzoquinone, are more water-soluble, but still sufficiently membrane permeable to function as transmembrane electron mediators.

Around the time that principles of transmembrane charge transfer were elucidated, photosynthetic principles of photoactive pigments were revealed by planar, bilayer or ‘black’ lipid membranes (BLM) experiments (Table 2, Figure 3b). In the most basic BLM experiment,^82^ a decane solution containing lipids and pigments is ‘painted’ over a small aperture in a hydrophobic, plastic material. The lipids self-assemble into a bilayer lipid membrane that spans the aperture and divides the two aqueous compartments. The electrical potential and/or current between electrodes, typically Ag/AgCl, either side of the membrane is then measured upon illumination and directly reports on charge transfer through the membrane.

**Table 2:** Early Studies on Light-Driven Transmembrane Charge Transfer^a^

Donor (if used)	Photosensitizer	Acceptor (if used)	Ref	Year
Various	photoactive pigments of chloroplasts	various	^84–90^	1968–1980
	xanthophylls and/or chlorophylls		^91^	1968
Fe^3+^	unknown		^92^,^93^	1971
	cyanine and other dyes		^94–97^	1972–1982
Fe(CN)_6_^4–^	various Mg-porphyrin variants	Fe(CN)_6_^3–^, O_2_	^98–102^	1972–1976
TMPD, Cytochrome c	chlorophyl *a*		^103^	1972
Fe(CN)_6_^4–^	chlorophyl *a*	Fe(CN)_6_^3–^	^83^	1977
PCu	C-phycocyanin		^104^	1979
	retinal, carotene and quinone covalently linked to porphyrin		^105^	1982
	aromatic amino acids		^106^	1982
AA	covalently linked porphyrin—quinone and other complexes	Fe^3+^	^107^	1982
AA	ZnTPP	Fe^3+^	^108^	1984
	Fullerene (C60)		^109,110^	1997, 2019

a For abbreviations and chemical formulas, see Figures 1 and 4. AA = ascorbic acid; TMPD= N,N,N′,N′-tetramethyl-p-phenylenediamine.

As the pigments are not specifically orientated with respect to the two compartments either side of the membrane, illumination alone does not lead to a significant transmembrane potential nor a net charge transport (electrical current). However, significant transmembrane currents were measured either by applying an external potential or by creating asymmetry in donor/acceptor molecules between the two compartments. For instance, Masters and Mauzerall dissolved ferro- and ferricyanide on either side of the membrane and measured the photocurrents through the BLM containing chlorophyll *a* as a function of applied transmembrane potential.^83^ The same study also measured the dark currents upon addition of quinone electron mediators such as plastoquinone. Besides natural pigments, BLM systems have been used to measure photoconductance across the membrane by C-phycocyanin protein, cyanine dyes, synthetic porphyrins, quinone-porphyrin compounds, aromatic amino acids and fullerenes, Table 2.

### 2.2 Photoinduced, Transmembrane Charge Transport with Redox Mediators

Following on from these early studies, vesicle-based photochemical systems were developed by combining vesicle-based systems, almost always liposomes, with photosensitizers, Table 3. Both water-soluble photosensitizers, Figure 3c, and hydrophobic, membrane localized photosensitizers, Figure 3b, have been extensively studied. Systems that used water-soluble photosensitizers necessarily employed electron mediators to transfer electrons across the membrane. For instance, Zamaraev et al. used Ru­(2,2’-bipyridine)_3_ (Ru­(bpy)_3_) as photosensitizer and ethylenediaminetetraacetic acid (EDTA) as sacrificial electron donor (both outside the vesicles), and used a hydrophobic viologen (C_16_V) as electron mediator to photoreduce ferricyanide in the lumen of the vesicles.^111^ Overall, only a limited number of water-soluble photosensitizers have been explored, typically either Ru­(bpy)_3_ or a water-soluble Zn-porphyrin, Table 3.

**Table 3:** Vesicle-Based Photochemical Systems with Water-Soluble or Membrane-Attached Photosensitizers^a^

Donor	Photosensitizer	Membrane Mediator	Acceptor	Comments	Ref	Year
EDTA	Proflavine	MQ MK	Fe(CN)_6_^3–^		^120^	1977
EDTA	di(C_16_) Ru(bpy)_3_^2+^	MK and C_16_V	MV		^112^	1978
EDTA	Ru(bpy)_3_	Ru(bpy)_3,_ MV^b^	C_7_V		^113^	1979
EDTA	di(C_16_) Ru(bpy)_3_	Ru(bpy)_3,_ MV^b^	C_7_V	Ionophore added for charge compensation	^114^	1981
EDTA	Ru(bpy)_3_	Ru(bpy)_3,_ MV^b^	MV	Showed that transmembrane ET is not possible without a mediator	^58^	1983
	ZnTMPyP					
potassium oxalate	ZnTMPyP	C_18_V			^121^	1986
EDTA	Ru(bpy)_3_	C_16_V	Fe(CN)_6_^3–^		^111^	1988
benzylalcohol	CdS quantum dots	C_16_V	Ag^+^		^115–117^	1988–1992
EDTA	Ru(bpy)_3_	1,4-bis(1,2,6-triphenyl-4-pyridyl)benzene	Pd → protons	Pd nanoparticles catalyze HER	^122^	1994
DTT	ZnTPPS	2,4,6-trimethylpyrylium,	Co(bpy)_3_^3+^		^123–125^	1998, 2000, 2001
		2,4,6-triphenylpyrylium, 2,4,6-triphenylthiopyrylium				
		1-carboxyethyl-4-cyanopyridinium				
		*N*-alkyl-4-cyanopyridinium				
EDTA	ZnTPPS	Linked Spiropyran-Anthraquinone	Co(bpy)_3_^3+^		^126^	2006
EDTA	Monododecane modified ZnTMPyP	MMP+	WST		^127^	2015
TEOA	Ru(bpy)_3_	MV	Pt colloids → proton	Pt colloids catalyze HER in an iCHELL compartment, in which the membrane is made of polyoxometalate (POM) anions and MV cations	^128^	2018

a For abbreviations and formulas, see Figure 4. bpy = 2,2’-bipyridine; di­(C_16_) Ru­(bpy)_3_^2+^ = N,N’-di­(1-hexadecyl)-2,2’ -bipyridine-4,4’ -dicarboxamide}-bis­(2,2’ -bipyridine)­ruthenium; DTT = dithiothreitol; EDTA = ethylenediaminetetraacetic acid; HER = hydrogen evolution reaction; MMP+ = 1-methoxy-*N*-methylphenazinium; TEOA = triethanolamine; WST = 2-(4-iodophenyl)-3-(4-nitrophenyl)-5-(2,4-disulfophenyl)-2H-tetrazolium anion.

b It was proposed Ru­(bpy)_3_ can transfer electrons across the membrane, but later papers^58–60^ indicate that reduced MV is able to diffuse through the membrane and likely was responsible for the observed transmembrane electron transfer.

Photosensitizers have also been attached to or incorporated into the lipid membrane by synthetically adding hydrophobic groups to the photosensitizer. In most cases electron mediators were still required for transmembrane electron transport suggesting that these photosensitizers do not freely diffuse through the hydrophobic core of the membrane. For instance, when Ford et al. attached Ru­(bpy)_3_ to the membrane by modifying one of the bpy-ligands with two alkane chains to give di­(C_16_)­Ru­(bpy)_3_^2+^, quinone mediators were required for transmembrane electron transfer.^112^ We note here that some early studies suggested that Ru­(bpy)_3_^2+^ or di­(C_16_)­Ru­(bpy)_3_^2+^ could mediate photoelectrons across the membrane,^113,114^ but these studies used viologens as electron acceptor. As mentioned earlier, it has since been shown that the monovalent viologen cation (e.g., MV^·+^) can pass electrons through a lipid membrane,^58–60^ possibly via disproportionation of two radicals,^61^ and were likely responsible for the observed photoinduced transmembrane charge transport in these earlier studies.

Besides Ru­(bpy)_3_ or Zn-porphyrin, quantum dots have been studied as photosensitizer.^115–117^ Horváth and Fendler synthesized CdS quantum dots from Cd^2+^ and H_2_S in the presence of lipid vesicles (dihexadecyl phosphate, DPH), resulting in 2.5–5 nm sized CdS nanoparticles.^115^ Cd^2+^ was present both inside and outside the vesicles, but only the smaller (2.5 – 4 nm) particles in the lumen of the vesicles, likely size-limited by the finite amount of encapsulated Cd^2+^, were found to be photoactive and able to transfer photoelectrons to a viologen mediator (C_16_V). The photoreduced C_16_V reduced Ag^+^ to metallic silver on the outside of the vesicles. Tricot and Manassen used the same method for CdS synthesis, but used vesicle preparations in which Cd^2+^ was either only present in the lumen or only present in the extravesicular solution.^116,117^ CdS was observed to only efficiently reduce MV^2+^ when MV was placed at the same side of the membrane as the CdS, confirming that the CdS quantum dots do not efficiently transverse a lipid membrane.

As summarized in Table 4, several groups have published approaches with hydrophobic photosensitizers localized in the lipid membrane. In these systems the photosensitizer acts as both light harvester and redox mediator, transferring charge across the membrane upon illumination, Figure 3b. As with the early BLM experiments, directionality of charge transfer is obtained by asymmetry in redox conditions between the lumen and extravesicular solutions, i.e. the electron donor and acceptor are placed at opposite sides of the vesicle membrane. Aboshi et al. attached a single pyrene group to a Ir­(bpy)_3_ photosensitizer and showed light-driven transmembrane electron transfer from ascobic acid in the lumen to extravesicular MV.^118^ Fluorescence lifetime analysis of the pyrene-modified Ir­(bpy)_3_ showed that the photoexcited Ir­(bpy)_3_ is quenched by the pyrene group and the authors proposed that, after oxidation of the photoexcited Ir­(bpy)_3_ by MV, the positive charge is localized at pyrene group. Aboshi et al. further hypothesized that transmembrane electron transfer is accomplished by charge transfer between two pyrene groups at either side of the membrane.^118^ A similar approach was reported by Kelson et al.^119^ who attached a naphthalene diimide group to a Zn-porphyrin photosensitizer. Interestingly, transmembrane photoelectron transfer was only observed when a longer diithiophene linker was used between the napthalene and porphyrin group. The authors proposed that the total length of the porphyrin-dithiophene-naphthanene triad was sufficient to transverse the membrane and that this geometry was responsble for its ability to support transmembrane photoelectron transfer. In this last example, the photosensitizer was thus hypothesized to form a conduit through the membrane, rather than diffuse across the membrane. Further discussion of transmembrane charge transfer conduits is provided in Section 3.

**Table 4:** Vesicle-Based Photochemical Systems with Membrane-Localized Photosensitizers^a^

Donor	Photosensitizer	Acceptor	Comment	Ref	Year
AA	methylene blue	Fe(CN)_6_^3–^		^129–131^	1979, 1985
Fe^2+^					
AA	phenosafranin, neutral red, thionine	Fe(CN)_6_^3–^		^132^,^133^	1980
AA	chlorophyllin *a*	Fe(CN)_6_^3–^		^134^,^135^	1980, 1983
AA	phenosafranine	Fe(CN)_6_^3–^		^136^	1983
EDTA					
EDTA	ZnTPP	MV → Proton	Hydrogenase or Rh-based catalyst for HER	^137^	1983
glutathione	chlorophyllin	MV		^138^	1985
AA	chlorophyll *a* pheophytin	Ethyl viologen sulfate	Hydrogenase catalyzes HER	^139^	1987
		MV → Proton			
EDTA	ZnTPP	MV		^111^	1988
AA	acridine orange	MV		^140^	1988
AA	fullerene (C70)	anthraquinone 2-sulfonate		^141^	1993
alkyl-pyrene	alkyl-pyrene	D_2_O	Alkyl-pyrene(+) radicals were detected by EPR spectroscopy.	^142^	1998
chlorophyll *a*	chlorophyll *a*	H_2_O	Chlorophyl(+) radicals were detected by EPR spectroscopy.	^143^,^144^ ^b^	2002, 2011
AA	1(hydroxymethyl)pyrene	MV		^145^	2011
AA	monopyrene substituted Ir(bpy)_3_	MV		^118^	2015
EDTA	Zinc-porphyrin-naphthalene diimide	MK		^119^	2015

a For abbreviations and formulas, see Figure 4. AA = ascorbic acid; EDTA = ethylenediaminetetraacetic acid; EPR = electron paramagnetic resonance; HER = hydrogen evolution reaction.

b Both papers report the same data.

### 2.3 Compartmentalized Photocatalysis with Redox Mediators

Several groups have extended vesicle-based systems having vectoral, transmembrane photoelectron transfer to include chemical catalysis through the principles illustrated in Figure 3b,c,d. Compartmentalized light-driven hydrogen evolution was shown with Pd nanoparticles, using an unusual lipophilic viologen, 1,4-bis­(1,2,6-triphenyl-4-pyridyl)­benzene (benzyl viologen, BV), Figure 5a.^122^ In a more recent example with Pt nanoparticle catalysts, Nakanishi et al. replaced the lipid vesicle with a compartment prepared from polyoxometalate (POM) anions and MV cations, creating a inorganic compartment which the authors coined iCHELL, Figure 5b.^128^ The compartment is formed by mixing high concentrations of the anionic POM and cationic MV^2+^, which precipitate, forming millimeter sized compartments.

**Figure 5:**
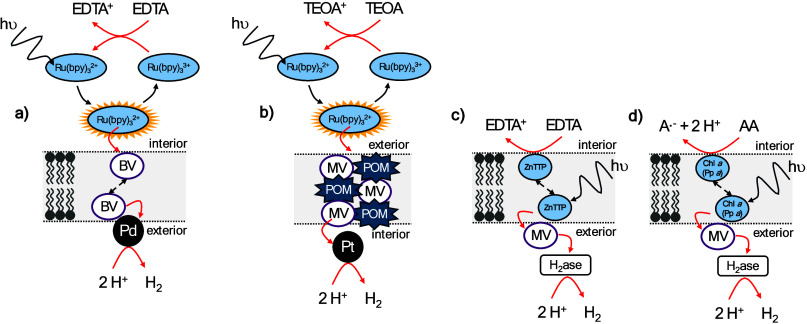
Schematic representation of the compartmentalized photocatalytic systems developed by (a) Efimova et al.,^122^ (b) Nakanishi et al.,^128^ (c) Tsvetkov et al.^137^ and (d) Semenova et al.^139^ AA = ascorbic acid; A·^–^ = monodehydroascorbate; BV = 1,4-bis­(1,2,6-triphenyl-4-pyridyl)­benzene; Chl *a* = chlorophyll *a*; EDTA= ethylenediaminetetraacetic acid; Pd = Palladium nanoparticles; MV = methyl viologen; POM = polyoxometalate ([PW_12_O_40_]^3–^; Pp *a* = pheophytin *a*; Pt = platinum nanoparticles; TEOA = triethanolamine; H_2_ase = hydrogenase from *Thiocapsa bogorovii* BBS (former name *T. roseopersicina*); ZnTTP = Zn 5,10,15,20-tetraphenyl-porphyrin.

The first biohybrid system, in which a vesicle-based photosynthetic system was combined with a biocatalyst was published in 1983, Figure 5c.^137^ The group of Lymar and Parmon combined a membrane-localized Zn-porphyrin photosensitizer with a hydrogenase from a purple sulfur bacterium for hydrogen evolution.^137^ The hydrogenase was added to the outside of vesicles, while the vesicles were loaded with EDTA that acted as the sacrificial electron donor (SED). Electron transfer from the Zn-porphyrin to the hydrogenase was mediated by MV and a quantum yield of 0.01% was achieved. Semenova et al. published a similar system in 1988,^139^ but instead used chlorophyl and pheophytin as photosensitizers, Figure 5d. Semenova et al. showed that a transmembrane potential, generated by transmembrane photoelectron transfer, ultimately limits the rate of photocatalysis. To disrupt the transmembrane potential valinomycin was added, which increased transmembrane photoelectron transfer and hydrogen formation by a factor of 1.5 to 2.^139^ Valinomycin is an ionophore that allows potassium cations to diffuse through the membrane, thereby disrupting electrochemical gradients in systems with potassium salts.

The work by Semenova et al. also highlights another principle. By adding the potassium ionophore, valinomycin, the photogenerated electrochemical potential was converted into a potassium ion gradient. Several groups have engineered vesicle systems that explored this principle further. The group of Gust, Moore and Moore synthesized an 8 nm long molecular triad (C–P-Q), which will be discussed in more detail in Section 3.1. By asymmetric incorporation of this triad into liposomes, a charge separated state was formed that spanned the membrane, forming a transmembrane potential. Quinones in the liposomal membrane then converted the transmembrane potential into proton transport, resulting a transmembrane pH gradient, Figure 3f.^146–150^ Hu et al. showed the same principle, but instead using a novel Janus metal–organic particles for transmembrane charge separation (see Section 3.1 for more details).^151^ The light-driven pH gradient generated by both systems were subsequently used to drive ATP synthesis.^150,151^ Noteworthy is that these systems mimic the principles of cyclic electron flow in chloroplast around PS I and cytochrome *b*_6_ *f*, Figure 1.^152^ Under cyclic electron flow, electrons are transferred from Fd to PQ, a plant mechanism thought to balance ATP/NADPH production and reduce photodamage under intense light conditions. This shunted electron transfer pathway produces a pH gradient without reducing NADP^+^ to NADPH, thus contributing to ATP synthesis without the oxidoreductive catalytic steps that lead to CO_2_ fixation. Finally, Gust, Moore and Moore showed that transmembrane potentials can also drive formation of gradients by cations other than protons, and used a Ca­(II) chelator to convert the transmembrane potential into a Ca­(II) gradient.^153^

## 3 Conduits for Light-Driven Transmembrane Charge-Transfer

Light-driven conduits performing transmembrane electron and ion transfer are the focus of this Section. Vesicles equipped with such conduits mimic the light-reactions of photosynthesis by transducing light energy into chemical energy in the form of a transmembrane redox gradient or concentration gradient. Redox gradients can be harnessed directly to form fuels through redox catalysis. Concentration gradients, particularly in the form of a *pmf*, can be harnessed to synthesize ATP which is a key intermediate for enzyme catalyzed synthesis of fuels and useful chemicals. A notable feature of several electron transfer conduits is that their photochemistry creates a redox gradient and produces a *pmf*.

### 3.1 Synthetic Redox-Active Conduits

Light-driven transmembrane electron transfer is integral to the function of PSI which couples the oxidation of PCu (*E*_m_ +0.37 V) to reduction of Fd (*E*_m_ −0.40 V) during photosynthesis, Figure 1. That photochemistry has inspired the design of synthetic molecules that mimic the functional properties of PSI with the general mechanism shown in Figure 3e. To couple the redox reactions of aqueous species on either side of a membrane, synthetic light-driven electron conduits can be symmetrical molecules having a hydrophobic region embedded in the membrane interior and terminal hydrophilic groups associated with the aqueous interfaces. Those molecules additionally act as molecular wires conducting electrons across the membrane in response to a phototrigger. An example from Sinambela et al.^154^ has a hydrophobic core comprised of fluorene and alkyne units linked to terminal redox-active bis­(triarylamine) units functionalized with carboxylic acids for hydrophilicity, Figure 6a. Functional properties were studied with this molecule embedded in liposomes where the directionality of electron transfer was enforced by placing water-soluble electron donor and acceptor molecules on opposite sides of the membrane, Figure 7a. Irradiation at 470 nm selectively excited the molecular wire triggering oxidation of NADH and reduction of XTT (2,3-bis­(2-methoxy-4-nitro-5-sulfophenyl)-2*H*-tetrazolium-5-carboxanilide). The authors proposed membrane-mediated electron transfer promoted via a mixed valence radical cation state of the one-electron oxidized molecular wire, delocalizing an unpaired electron across the entire molecule. Rates of light-driven electron transfer were faster with XTT encapsulated, and the liposomes surrounded by NADH. This observation was attributed to a rate limiting oxidation of XTT that was accelerated by its encapsulation within the liposome.

**Figure 6:**
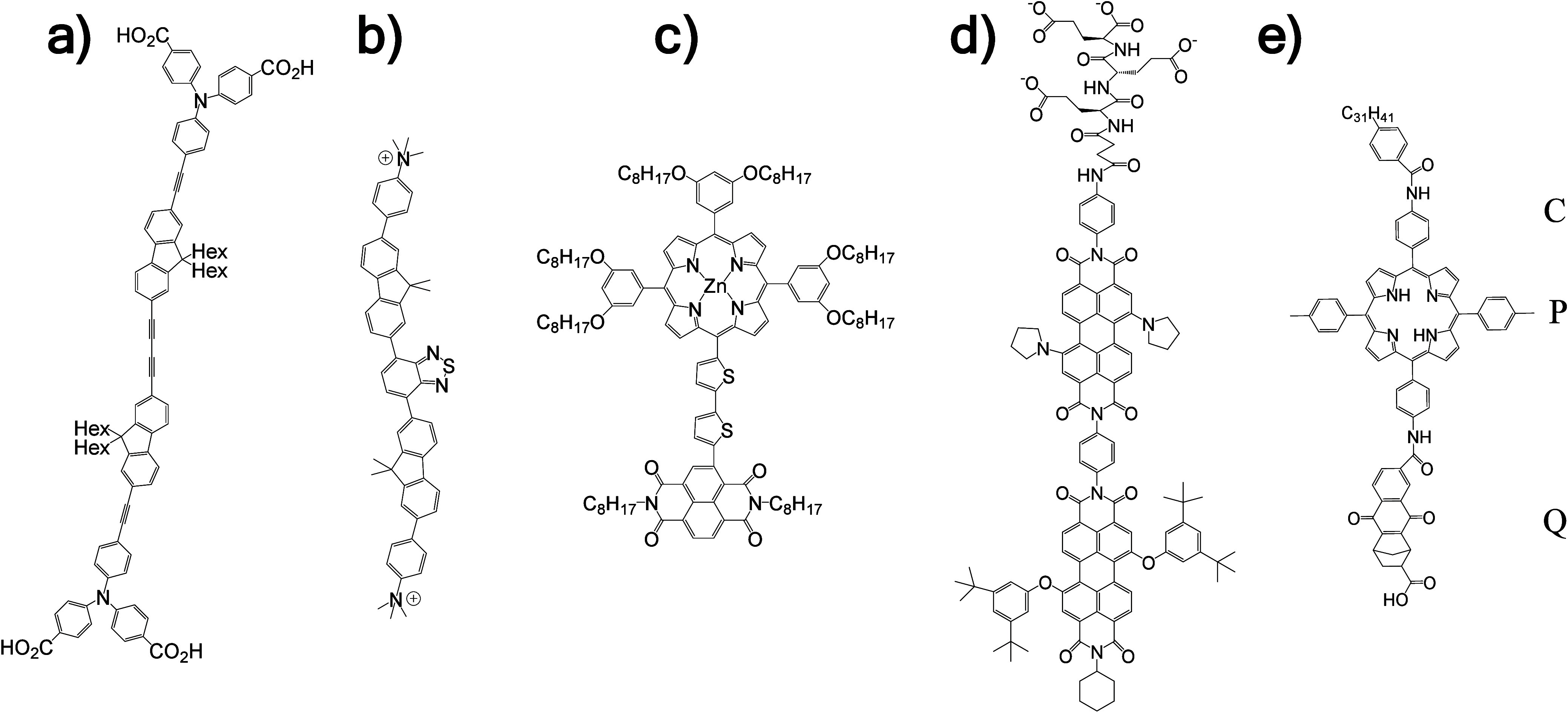
Synthetic redox conduits developed by (a) Sinambela et al.,^154^ (b) Sinambela et al.,^155^ (c) Kelson et al.,^119^ (d) Perez-Velasco et al.^156^ and (e) Steinberg-Yfrach et al.^149^ C = carotenoid polyene; Hex = nonpolar aliphatic hexyl tail; P = tetraarylporphyrin; Q = naphthoquinone.

**Figure 7:**
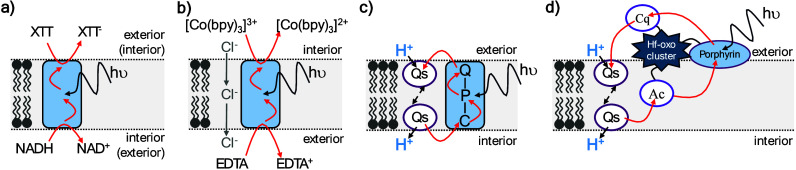
Schematic representations of the compartmentalized photocatalytic systems developed by (a) Sinambela et al.,^154^ (b) Perez-Velasco et al.,^156^ (c) Steinberg-Yfrach et al.^149^ and (d) Hu et al.^151^ Ac = acitretin; bpy = bipyridine; C = carotenoid polyene; Cq = carboxyquinone; EDTA = ethylenediaminetetraacetic acid; NADH = dihydronicotinamide adenine dinucleotide; P = tetraarylporphyrin; Q = naphthoquinone; Qs = diffusible quinone; XTT = 2,3-bis­(2-methoxy-4-nitro-5-sulfophenyl)-2H-tetrazolium-5-carboxanilide.

Similar photochemistry was displayed by a second light-activated molecular wire described by Sinambela et al.^155^ In that molecule, Figure 6b, a central benzothiadiazole absorbed visible light, fluorene provided hydrophobicity and terminal trimethylammonium groups hydrophilicity. Light-driven electron transfer from NADH to XTT was demonstrated with this molecule embedded in liposome bilayers and the authors proposed two possible photocycles. The photoexcited molecular wire may experience reductive quenching through NADH oxidation followed by a return to the ground state through XTT reduction. Alternatively, oxidative quenching by XTT reduction would be followed by a return to the ground state through NADH oxidation. Of note is that this light-driven conduit operates in both aerobic and anaerobic atmospheres. Thus, there is limited quenching of the photoinduced electron transfer reactions in the presence of molecular dioxygen.

Another symmetrical molecule performing phototriggered electron transfer is the light-harvesting conjugated oligoelectrolyte reported by Chen et al.^157^ That molecule contained a conjugated acceptor–donor–acceptor backbone to facilitate charge separation following excitation with visible light. When intercalated into lipid bilayers, the molecule enabled photocatalytic N_2_-fixation in a bacterial cell since the reducing potential of the excited state (−0.85 V) was sufficient to allow reduction of flavodoxin and/or Fd (*E*_m_ −0.46 V) and eventually the enzyme nitrogenase that catalyzed N_2_-fixation. Ascorbate provided the SED.

Photocatalytic transmembrane electron transfer by a donor–acceptor dyad was described by Kelson et al.^119^ The dyad was comprised of naphthalene diimide as electron acceptor and zinc-porphyrin as both light absorber and electron donor, Figure 6c. The dyad had a length of approximately 3.5 nm which provided a good match to the thickness of a standard lipid bilayer membrane. Long alkyl chains at the periphery of the porphyrin provide hydrophobicity. When irradiated in lipid bilayers the dyad enabled reduction of encapsulated water-soluble naphthoquinone. Naphthoquinone reduction was accompanied by protonation, lowering the internal proton concentration such that transmembrane electron transfer was accompanied by *pmf* formation. Oxidation of externally added EDTA returned the ground state zinc-porphyrin photosensitizer. Bhosale et al.^158^ coupled light-driven redox chemistry on opposite sides of a membrane using self-organizing helical tetrameric π-stacks of fluorescent naphthalene diimides supported on rigid *p*-octiphenyl rods. The diimides carried electron donating alkylamine substituents that promoted photoinduced charged separation. When embedded in lipid vesicles and irradiated with visible light these supramolecular assemblies oxidized external EDTA and reduced quinone at their internal surface, again coupling transmembrane electron transfer to *pmf* formation. Also reported are molecular wires that spontaneously transport electrons across lipid bilayers in response to the driving force imposed by redox partners on either side of that membrane.^159,160^ These electron conduits could be harnessed for solar chemicals production by partnering them with appropriate photosensitizers, for example as illustrated conceptually in Figure 3d.

As noted in Section 2.3, uncompensated unidirectional movement of electrons across a membrane results in a buildup of transmembrane potential that will slow down, and ultimately stop, further electron transfer. When addition of a molecule dissipating the transmembrane potential leads to enhanced electron transfer it is good evidence to support the proposed mechanism of electron transfer.^119,149,155,158^ In an alternative approach to avoid inhibitory buildup of transmembrane potential, Perez-Velasco et al.^156^ designed conduits that performed electroneutral electron–anion antiport. An oligo­(p-phenylene)-N,N-perylenediimide (O-PDI) rod included the green 1,7-bis­(pyrrolidin-1-yl)-3,4:9,10-perylene-bis­(dicarboximide) chromophore for light-absorption and charge separation in the inner leaflet of the bilayer membrane, Figure 6d. Anionic triglutamate tails at one end of the rod provided hydrophilicity that drove unidirectional insertion into preformed liposomes. Subsequent irradiation resulted in reduction of encapsulated [Co­(bpy)_3_]^3+^ coupled to oxidation of external EDTA. Photocatalytic rates measured in the presence and absence of molecules that dissipated the transmembrane potential were nearly superimposable. Thus, light-triggered transmembrane transport by the O-PDI rod is electroneutral with active electron influx accompanied by passive anion efflux. The latter was allowed by anion−π interactions along the π-acidic backbones of the O-PDI rods, Figure 7b. Similar behavior was described for an O-PDI rod that included red chromophores with two phenoxy rather than two pyrrolidinyl substituents in the core.^156^

The aforementioned systems achieve directionality of transmembrane electron transfer by virtue of the location of the spatially separated electron donors and acceptors. Other molecules impose the direction of electron transfer through their asymmetry. For example, photosensitizers anchored asymmetrically in a membrane by appropriate hydrophobic groups can generate charge separated states in which the photoenergized electron and hole are localized to opposite sides of the membrane, Figure 3f. Steinberg-Yfrach et al.^149^ reported a molecular triad comprised of photosensitive tetraarylporphyrin (P) linked to both an electron donor and an electron acceptor, Figure 6e. The electron donor was a carotenoid polyene (C) and the electron acceptor a naphthoquinone fused to a norbornene system bearing a carboxylic acid group (Q). Photoexcitation produced the porphyrin singlet excited state from which internal electron transfer led to formation of the charge separated species, C-P^+^-Q^–^. Subsequent electron transfer from the carotenoid to the porphyrin radical cation competed with charge recombination to give C^+^-P-Q^–^ with a quantum yield up to 0.15. To avoid the energetic cost of moving the polar, carboxylate-bearing quinone (Q) through the hydrophobic membrane interior, the triad inserted into preformed liposomes with a single orientation having C facing inside and Q outside, Figure 7c. As a consequence, the photogenerated C^+^-P-Q^–^ state placed a reductant near the outer surface of the bilayer and an oxidant near the inner surface. When a membrane soluble quinone was included, that molecule was reduced at the outer membrane surface in a process accompanied by proton uptake, Figure 7c. Subsequent quinol oxidation was catalyzed at the inner membrane surface and released protons to the liposome interior. The process had an overall quantum yield of approximately 0.04 and the proposed mechanism was supported by acidification of the liposome interior such that *pmf* creation again accompanied transmembrane electron transfer.

Hu et al.^151^ described a light-driven electron conduit having multiple functional groups structured around Hf-oxo clusters (Hf_6_(μ_3_-O)_4_(μ_3_–OH)_4_) which they termed Janus metal–organic layers, Figure 7d. Acitretin provided a hydrophobic hole acceptor that inserted into the bilayer of preformed liposomes. Hydrophilic components to associate with the external membrane surface were provided by a light-harvesting porphyrin and electron accepting carboxyquinone. Following photoexcitation of the porphyrin, the energized electron located to the carboxyquinone and the hole to the membrane embedded acitretin. The spatially resolved redox sites then drove redox cycling of a lipid soluble diffusible quinone leading to vectorial translocation of protons across the lipid bilayer, Figure 7d. The apparent quantum efficiency of proton transport was estimated as 0.07% with an upper limit for the intrinsic proton transport rate of approximately 14 s^–1^ porphyrin^–1^.

Unidirectional light-driven transmembrane electron transfer in the presence and absence of dioxygen has also been reported for the metallopeptide designed by Klein et al.^161^ The peptide sequence was based on that of artificial hydrophobic α-helical peptides developed as models for the hydrophobic domains of naturally occurring transmembrane proteins. The unnatural amino acid, bipyridylalanine, was introduced at two locations to allow chelation of two [Re­(I)­(CO)_3_Cl] units on either side of the bilayer. That resulted in a neutral metallopeptide with the inorganic centers providing both photosensitizers and redox sites. Thus, when embedded in liposomes, transmembrane photoinduced electron transfer was facilitated by splitting one long (hence infinitely slow) electron transfer step, into three shorter and thus faster steps, Figure 8a mechanism I. The same peptide functionalized with two [Ru­(II)­(bpy)_2_] centers carried a 4+ charge.^161^ When introduced to liposomes and irradiated, the [Ru­(II)­(bpy)_2_]_2_-peptide caused electron donor molecules to leak through the lipid bilayer to the liposome exterior. Those donor molecules, once outside the liposomes, were oxidized in a light-dependent manner by [Ru­(II)­(bpy)_2_]_2_-peptides associated with the outer membrane surface, Figure 8a mechanism II. The results highlighted the need for careful experimentation to validate the processes occurring in irradiated liposomes.

**Figure 8:**
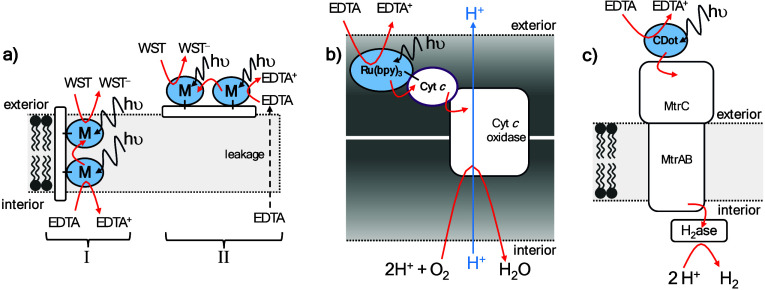
Schematic representations of metallopeptide and metalloprotein conduits for light-driven transmembrane electron transfer reported by (a) Klein et al.,^161^ (b) Hvasanov et al.^162^ and (c) Zhang et al.^163^ In a) the mechanism I is for M = Re­(I)­(CO)­Cl and mechanism II is for M = Ru­(II)­(bpy)_2_. bpy = bipyridine; Cyt *c* = cytochrome *c*; Cyt *c* oxidase = cytochrome *c* oxidase; EDTA = ethylenediaminetetraacetic acid; H_2_ase = [FeFe]-hydrogenase from *Clostridium beijerinckii*; M = Re­(I)­(CO)Cl and Ru­(II)­(bpy)_2_; MtrAB = outer membrane spanning porin-cytochrome complex of *S. oneidensis*; MtrC = extracellular cytochrome of *S. oneidensis*; WST = 2-(4-iodophenyl)-3-(4-nitrophenyl)-5-(2,4-disulfophenyl)-2*H*-tetrazolium anion.

### 3.2 Biological Redox-Active Conduits

PSI and PSII naturally perform light-driven electron transfer across lipid bilayers as shown schematically in Figure 3e and Figure 3g, respectively. Although nearly 100% quantum efficiency is achieved during the primary process, light harvested by these enzymes is constrained to the visible range which means there is relatively low energy conversion of incident light available from the solar spectrum.^4,164,165^ To address this situation, synthetic materials can be included that harvest photons from a wider range of wavelengths and mimic the role of the LHCs in natural photosynthesis, Figure 1. Xu et al.^166^ employed CuInS_2_/ZnS quantum dots to funnel energy from ultraviolet light to PSII and enhance rates of transmembrane electron transfer due to water oxidation, Figure 9a. Those quantum dots exhibited a large Stokes shift such that their excitation by ultraviolet light is followed by emission of red light centered around 680 nm where PSII has maximal absorbance. A similar conversion was achieved by Wang et al.^167^ with conjugated polymer nanoparticles. Increased electron transport through PSI and PSII was achieved by Zhou et al. with a cationic poly­(fluorene-*co*-phenylene) conducting polymer.^168^ The positively charged side chains of the polymer promoted electrostatic binding to lipid bilayers. The intense broad absorption band of the polymer, from 300 to 420 nm, was complementary to that of the enzymes. Maximum polymer emission was at 425 nm which overlapped with enzyme absorbance. Another study^169^ used phycocyanin, a water-soluble pigment, to effect energy transfer to PSII since the emission peak of phycocyanin at 642 nm matched the 650 nm absorption peak of PSII. With the ratio of phycocyanin to PSII as 15:1 (μg:μg) the rate of transmembrane electron transfer is almost 2-fold that in the absence of phycocyanin. Other approaches to increased light harvesting used CdTe quantum dots^170^ and nanotubular titania.^171^

**Figure 9:**
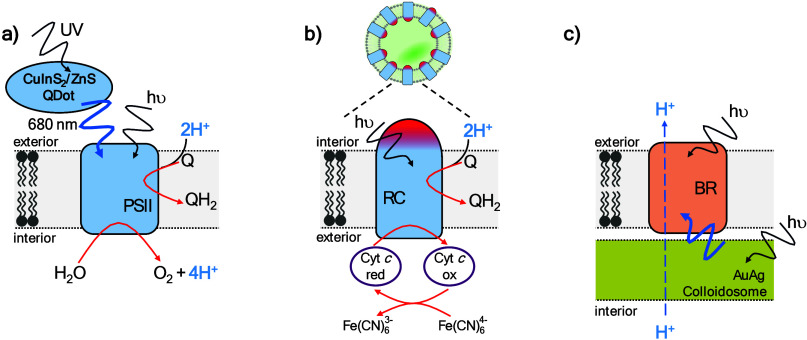
Schematic representation of methods that enhance the photoactivity of protein redox conduits as reported by (a) Xu et al.,^166^ (b) Altamura et al.,^179^ and (c) Chen et al.^203^ BR = bacteriorhodopsin; Cyt *c* (red/ox) = cytochrome *c* (reduced/oxidized); PSII = photosystem II; RC = reaction center.

The performance of PSI and PSII can also be enhanced by their incorporation in polymer membranes. PSII reconstituted in vesicles comprised of synthetic phytanyl chained glycolipid showed 5- to 6-fold higher activity than PSII in phosphatidylcholine liposomes.^172^ Photocurrents from PSI hosted in membranes formed of the block copolymer poly­(butadiene)_12_-poly­(ethylene oxide)_8_ were stable for at least 1 month.^160^ PSI wiring to a metal electrode was achieved with an underlayer of the same block copolymer made conductive by the presence of an intercalated conjugated oligoelectrolyte.

Another natural family of light-activated electron conduits is comprised of the photosynthetic reaction centers (RCs) extracted from species of *Rhodobacter* and *Rhodopseudomonas*.^173,174^ Photoexcitation of RCs generates an electron–hole couple that oxidizes water-soluble cytochrome *c*_2_ on one side of the membrane and reduces ubiquinone on the other side of the membrane, Figure 3g. Several studies^175–179^ have reproduced that activity in liposomes hosting a quinone as electron acceptor and externally added reduced cytochrome *c*, sometimes supplemented with potassium ferrocyanide, as electron donor, Figure 9b. The promiscuity of cytochrome *c* and potassium ferrocyanide as redox partners, see for example^180–182^ , should enable this system to be coupled with synthetic catalysts for semiartificial photosynthesis. Of note is the extremely uniform orientation (90 ± 1%) of RCs incorporated into liposomes of approximately 20 μm diameter by Altamura et al.^179^ This was achieved with a droplet transfer method whereby a homogeneous micellar solution of RCs was emulsified in mineral oil containing a mixture of phosphatidylcholine and phosphatidylglycerol. The emulsion was then layered on an aqueous solution, generating a biphasic system from which RC containing liposomes were obtained by centrifugation. The directionality achieved by Altamura et al. is in contrast to earlier studies which report incorporation that is essentially random^175,178,183–187^ or with a small bias to one orientation.^178^

Several membrane spanning proteins transfer electrons spontaneously across lipid bilayers in processes driven by the relative reduction potentials of spatially separated redox partners. When incorporated into vesicles, such conduits can in principle partner with photosensitizers to enable light-driven electron exchange with encapsulated catalysts for semiartificial photosynthesis, for example as shown in Figure 3d. To initiate light-driven transmembrane electron transfer across a polymersome membrane, Hvasanov et al.^162^ used cytochrome *c* oxidase as the membrane spanning redox-active conduit. In mitochondria, cytochrome *c* oxidase creates a transmembrane pH gradient in response to coupling the oxidation of water-soluble cytochrome *c* to reduction of O_2_ on the opposite side of the mitochondrial membrane. In the experiments of Hvasanov et al., photoenergized electrons were introduced to cytochrome *c* oxidase from its natural redox partner cytochrome *c* that was photosensitized by covalent attachment of Ru­(II)-bis­(terpyridine), Figure 8b. Vesicles were prepared from polystyrene_140_-*b*-poly­(acrylic acid)_48_ diblock copolymer with simultaneous encapsulation of the photosensitized cytochrome *c* and cytochrome *c* oxidase. The polymer membrane was considerably thicker (98 ± 35 nm) than natural phospholipid bilayers and the authors proposed selective encapsulation of photosensitized cytochrome *c* in the hydrophilic poly­(acrylic acid)_48_ block and cytochrome *c* oxidase in the hydrophobic polystyrene_140_ block due to electrostatic interactions, Figure 8b. Photoexcitation of the Ru­(II) dye photosensitizer in the presence of sacrificial electron donor EDTA in the external solution led to a pH increase inside the vesicles. The pH gradient provided evidence for light-driven electron transfer from external EDTA to internal O_2_ supported by redox cycling of cytochrome *c* and cytochrome *c* oxidase.

More recently, Zhang et al.^163^ used a redox active protein conduit to achieve semiartificial photosynthetic H_2_ production by encapsulated hydrogenase enzyme, Figure 8c. Photoenergized electrons were conducted across the liposome membrane by an electron conduit formed by a complex of three tightly bound proteins, MtrCAB. The MtrCAB complex contains a chain of 20 close-packed redox-active heme cofactors^188^ that move electrons across the membrane through complementary Fe­(III) ↔ Fe­(II) transitions of neighboring sites. MtrCAB reconstituted into liposome bilayers supported transmembrane electron transfer at rates approaching 10 000 *e* ^–^ s^–1^ MtrCAB^–1^ dependent on the nature of the chemical donor and acceptor species.^189^ Adding graphitic nitrogen-doped carbon dots (g-N-CDs) as external photosensitizers allowed photoreduction of MtrCAB liposomes and H_2_-evolution by internalized hydrogenase.^163,190,191^ Hemes positioned close to the surface of MtrCAB on either side of the membrane spanning core allow electron transfer with appropriate partners^188^ and there was direct electron transfer from MtrCAB to the hydrogenase enzyme.^163^ In a complementary study, Piper et al.^191^ required encapsulated methyl viologen to shuttle photoenergized electrons from MtrCAB to coencapsulated nitrous oxide reductase. Given that many proteins and synthetic catalysts exchange electrons with methyl viologen, for example^45^,^137^,^139^,^192–195^ , several different photoproducts should be accessible from appropriately loaded MtrCAB carrying vesicles.

An important difference can be made in using naturally photoactive proteins, e.g., PSI, PSII and RC, and protein wires paired with photosensitizers. For the naturally photoactive proteins the direction of electron transfer across the membrane is dependent on the orientation of the protein in that membrane, and the locations of the relevant electron donor and acceptor. Where protein wires are paired with photosensitizers, the direction of light-induced electron transfer is typically imposed by the location of the relevant donor and acceptor, i.e., productive photocatalysis may not require the conduits to adopt a single orientation in the membrane. This contrasts with the situation found in biology where the proteins take a single orientation for cellular function. Other proteins that serve as redox conduits in biology could also be harnessed in biohybrid vesicles to take a similar role to MtrCAB. These include porin-cytochrome complexes that are homologues of Mtr­(C)­AB which span bacterial outer membranes to transfer electrons between cellular proteins and external partners including transition metal (oxy)­hydroxide particles and electrodes.^196–199^ Proteins in the cytochrome *b*_561_ family of diheme cytochromes that transfer electrons from cytoplasmic ascorbate to electron acceptors on the noncytoplasmic side of the membrane.^200,201^ We anticipate proteins developed through *de novo* design will also be valuable additions to the redox conduits available for study.^202^

### 3.3 Conduits for Proton Transfer

As noted above, PSII creates a *pmf* across thylakoid membranes in a light-driven process for which the steps are tightly coordinated with electron transfer.^204^ However, this elegant chemistry is performed within a fragile multiprotein supercomplex which presents challenges for studying PSII and developing associated technology. As a consequence, and as summarized in the review by Wang et al.,^205^ studies requiring light-driven proton conduits are typically performed with the much simpler and more robust bacteriorhodopsins (BRs). These proteins are the smallest light-driven proton conduits known in biology.^206–211^ BRs adopt a single orientation in the membrane where they are clustered as hexamers of purple color due to the presence of retinal chromophores. On photoexcitation, the chromophore undergoes a conformational change that translocates one proton across the membrane.^210,211^ Thus, the mechanism of *pmf* creation by BR, Figure 3h, is fundamentally different to that operating in PSII, Figure 3g.

Most widely studied in vesicles is BR from *Halobacterium salinarium*, formerly known as *H. halobium*, either as the purified monomer or as purple membrane patches.^206–211^ The latter are highly ordered native patches isolated from bacterial membranes and comprised of BR hexamers with specific lipids arranged in a crystalline lattice. Since the first demonstrations of light-induced proton transfer in BR containing vesicles, the proton pumping productivity has been improved by variations to the assembly procedure, shell composition and choice of enzyme. Several factors are key to effective operation. For example, the number of active pumps and their orientation with respect to the membrane, the passive permeability of the membrane, and the back-pressure effects associated with creation of ΔΨ and ΔpH that inhibit proton pumping. Quantified improvements from across the literature are hard to provide due to the many different formats for which the information is provided. The review of Rigaud et al.^212^ tabulates some relevant materials in their Table 4. In Table 5 we summarize key information from articles with particularly insightful studies in this area with the most biohybrid relevant approaches noted below.

**Table 5:** A Selection of Literature on Light-Triggered *pmf* Formation by BR and Purple Membranes Relevant to the Topic of This Review^a^

	H^+^ transport	Comments	Ref	Year
Liposomes	in	Purple membranes reconstituted in liposomes taking up protons (50 to 200 ng of protons per mg of purple protein). Addition of ionophore valinomycin accelerated the rate of uptake. With ATP synthase included the vesicles catalyzed light-dependent phosphorylation.	^218^	1974
	in	Purple membranes reconstituted in liposomes taking up protons (65 equivalents of protons per mg of BR).	^219^	1975
	in	Maximal accumulation of protons per BR molecule are tabulated for different liposome assembly methods.	^220^	1982
	in *and* out	Light-induced proton translocation was measured for BR containing liposomes. From the effect of the one-sided inhibitor La^3+^, which binds to the C-terminal surface where H^+^ enter BR, the presence of BR in both orientations was demonstrated,	^221^	1985
	in	Light-induced proton uptake by BR containing liposomes studied in response to the presence of ionophores, variation of actinic light intensity and lipid to protein ratio of the vesicles. The pH gradient across the membrane was increased to 2 units in the presence of valinomycin. Light-induced steady-state proton electrochemical potential was only partially determined by the amount of BR and proton passive permeability. Back-pressure effects were found to be strong regulating factors.	^222^	1986
	In *and* out	Reconstitution of BR into liposomes produced both inwardly proton pumping and outwardly proton pumping vesicles. The proportion of outwardly pumping liposomes increased with decreasing liposome size. The larger liposomes (diameter >200 nm) were shown to be pure inwardly pumping liposomes with almost homogeneous BR orientation.	^223^	1988
	in	BR was inserted into preformed liposomes. Experiments with La^3+^, that binds to the BR C-terminal surface where H^+^ enter BR, indicated that the inside-out configuration dominates. This is consistent with light-dependent decrease of internal pH by approximately 0.6 units.	^224^	2005
		Proteogels containing BR proteoliposomes exhibit a stable proton gradient when irradiated with visible light, whereas proteogels containing proteoliposomes with both BR and ATP synthase couple the photoinduced proton gradient to the production of ATP.		
	in *and* out	Manipulation of lipid composition defined the surface charge of liposomes and controlled the orientation of asymmetrically charged proteorhodopsin reconstituted into those liposomes.	^217^	2013
	out	Colloidosomes coated with liposomes comprised of purple membranes when irradiated acidify the external solution. The AgAuNP colloidosomes mimic the role of LHCs in natural photosynthesis and enhance light-dependent proton transfer. Including these vesicles in a solution of independently prepared ATP synthase containing liposomes enabled light-dependent ATP synthesis inside those liposomes.	^203^	2019
Polymersomes and Hybrid Vesicles	in *and* out	A series of papers that demonstrate how the direction of H^+^ translocation in vesicles prepared with the ABA triblock copolymer PEtOz–PDMS–PEtOz depends on the assembly protocol and source of BR (purified BR, purple membranes prepared in house, commercially sourced purple membranes).	^213^,^214^,^216^	2005
				2006
				2007
	in	Proton transfer into BR-containing polymer/polymer and polymer/lipid vesicles was much less than for BR-containing liposomes. Polymer/lipid vesicles were 70/30 (mol %) PDMS/phosphatidyl choline and 50/50 PBd-*b*-PEG/phosphatidylcholine. Mixed polymer vesicles were 50/50 PDMS/PBd-*b*-PEG.	^43^	2020

a Abbreviations: LHC = light-harvesting complex; PBd-*b*-PEG = polybutadiene_22_-*b*-poly­(ethylene oxide)_14_; PDMS = poly­(dimethylsiloxane)-*g*-poly­(ethylene oxide); PEtOz–PDMS–PEtOz = [poly­(2-ethyl-2-oxazoline)-*b*-poly­(dimethylsiloxane)-*b*-poly­(2-ethyl-2-oxazoline)].

Chen et al. enhanced proton pumping through BR containing vesicles using the plasmonic effect of noble metal colloidal particles.^203^ Sealed colloidosome vesicles were formed by coating positively charged colloidosomes prepared from AuAg nanorods (NRs) with negatively charged purple membranes, Figure 9c. Continuous irradiation decreased pH in the external medium at a rate approximately 3-fold greater than for vesicles prepared around colloidosomes formed of Au and SiO_2_ nanoparticles. The authors attributed this behavior to the broad plasmon resonance of AuAgNRs that overlapped well with the absorption spectra of BR intermediates. Excitation of the ground state by green light induces retinal isomerization to initiate the photocycle. A long-lived intermediate (M412) can be returned to the ground state by blue light that triggers a bypass of the normal thermal decay. AuAgNRs had a broad surface plasmon resonance absorbance that covered the entire visible range, whereas AuNPs had strong absorption only above 500 nm and SiO_2_ NPs had no surface plasmon resonance.

It is notable that most reports of BR containing vesicles describe acidification of the internal aqueous chamber when irradiated, Table 5. Since the direction of proton translocation is an important consideration when the *pmf* is to be harnessed for subsequent chemical catalysis, we highlight here protocols affording control over the direction of proton translocation. In a series of reports, Choi et al. characterized vesicles prepared by combining a triblock copolymer with BR and purple membrane patches.^44,213–216^ The ABA copolymer was PEtOz–PDMS–PEtOz [poly­(2-ethyl-2-oxazoline)-*b*-poly­(dimethylsiloxane)-*b*-poly­(2-ethyl-2-oxazoline)] which provided a hydrophobic wall approximately 4 nm thick. The direction of light-driven proton translocation with vesicles that contained purple membrane patches was dependent on the solvent used to dissolve the block copolymer, Figure 10a.^214^ The direction of proton translocation was also dependent on whether BR monomers or purple membrane patches were employed^214^ and whether purple membrane patches were commercially sourced or prepared in house.^216^

**Figure 10:**
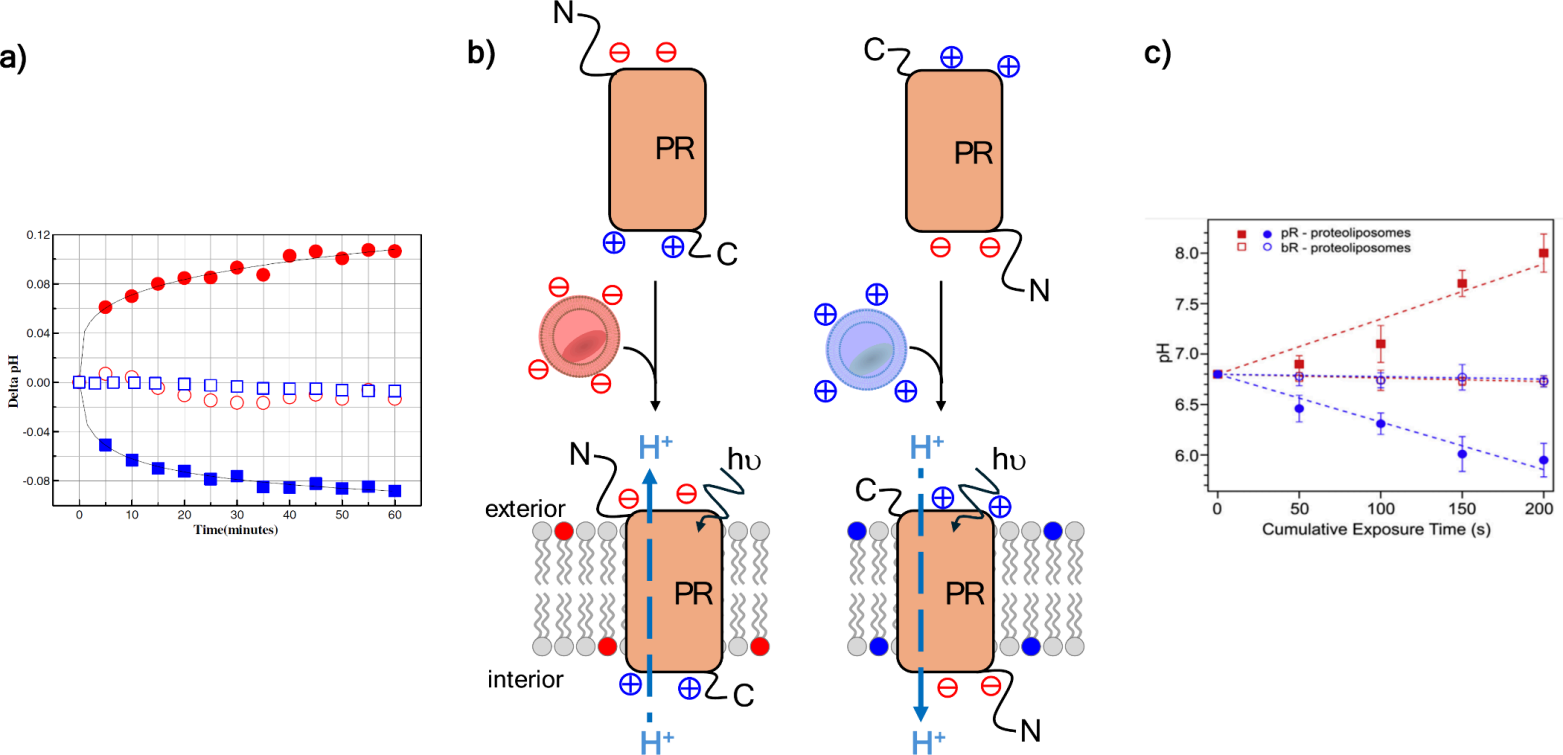
Imposing control over the direction of light-driven proton translocation as reported by (a) Choi et al.,^214^ and (b,c) Tunuguntla et al.^217^ (a) pH change within vesicles comprised of PEtOz–PDMS–PEtOz and purple membranes: prepared from ethanolic polymer solution and illuminated (■) or dark-incubated (□), prepared from polymer water mixture and illuminated (●) or dark-incubated (○). (b) Schematic showing asymmetrically charged proteorhodopsin approaching the charged surface of an anionic liposome (left) and a cationic liposome (right) as reported by Tunuguntla et al.^217^ and (c) the resultant pH change within irradiated anionic liposomes (red) and cationic liposomes (blue) that hosted pR (proteorhodopsin, filled symbols) and BR (open symbols). BR (bR) = bacteriorhodopsin; PEtOz–PDMS–PEtOz = [poly­(2-ethyl-2-oxazoline)-*b*-poly­(dimethylsiloxane)-*b*-poly­(2-ethyl-2-oxazoline)]; PR (pR) = proteorhodopsin. Panel a) Adapted with permission from ref 214. Copyright 2016, IOP Publishing. Panel c) Adapted with permission from ref 217. Copyright 2013, Cell Press.

Tunuguntla et al. described how the direction of proton pumping can be controlled by liposome surface charge.^217^ Their study employed anionic and cationic liposomes with an asymmetrically charged rhodopsin protein. That protein was proteorhodopsin which has approximately 24% sequence identity to BR. However, proteorhodopsin has an abundance of positively charged residues at the C-terminus and an abundance of negatively charged residues at the N-terminus, Figure 10b. Irradiation of proteorhodopsin inserted into anionic liposomes resulted in an increase in the lumen pH whereas the opposite trend was observed when cationic liposomes were used, Figure 10c. These observations were consistent with proteorhodopsin insertion into anionic liposomes driven by interactions with the protein C-terminus and insertion into cationic liposomes driven by interactions with the N-terminus. Equivalent experiments with BR failed to detect proton translocation, Figure 10c. The results were taken to indicate that BR, which lacks charge asymmetry, inserted into these liposomes randomly, i.e., with no preferred orientation.

## 4 Light-Driven ATP Synthesis and C-Fixation

ATP plays a critical role in biology as an energy carrying molecule and cosubstrate in many enzyme catalyzed reactions. As a consequence, harnessing photocatalytic processes for ATP synthesis presents an attractive way to access carbon-based fuels through biohybrid methodologies. For example, the critical role of ATP in biological C-fixation has stimulated much interest in powering artificial reaction cascades through light-driven ATP synthesis, i.e., photophosphorylation. In biohybrid vesicles, a photogenerated *pmf* is well suited for ATP synthesis, and such cascades typically employ F_0_F_1_-ATP synthase, here simply ATP synthase, for ATP synthesis. This enzyme produces ATP from ADP and inorganic phosphate (Pi) using energy derived from a transmembrane *pmf* as illustrated for the thylakoid membranes of green plants in Figure 1. By defining the spatially separated regions essential for *pmf* formation, vesicles hosting ATP synthase provide an obvious route to enable photophosphorylation and subsequent C-fixation. Interfacing such vesicles with synthetic molecules and materials, and perhaps with biological components from different organisms, may afford novel and energy efficient routes to solar-to-fuels conversion. Studies relevant to such approaches are the focus of this Section.

Some additional information about ATP synthase is warranted as a prelude to discussion of the vesicular systems. Functionally, ATP synthase is best considered as two main constituents: a hydrophobic membrane embedded F_0_ portion that allows proton translocation and a hydrophilic F_1_ portion that holds the binding sites for ADP and Pi and their conversion to ATP.^225–227^ In response to the *pmf*, protons move spontaneously across the membrane by entering the F_0_ portion, moving toward the F_1_ portion and then exiting the enzyme on the opposite side of the membrane to which they entered, Figure 3i. That flow of protons drives conformational change within F_1_ and in turn the binding of ADP and Pi followed by the formation and release of ATP. The structure of ATP synthase means that when the enzyme is incorporated into preformed vesicles it will insert hydrophobic F_0_ into the membrane with hydrophilic F_1_ protruding to the external solution. Consequently, most studies with vesicular ATP synthase report ATP synthesis in the external solution arising from acidification of the vesicle interior. A variety of photoconverters have been used to establish a *pmf* for ATP synthesis. Section 4.1 considers ATP synthesis driven by the *pmf* created by compartmentalized phototriggers. Section 4.2 describes ATP synthesis and carbon-fixation driven by the pmf created by light-driven charge transfer conduits and where successful catalysis depends on the correct localization and orientation of multiple components. For comprehensive reviews of ATP synthase reconstitution into liposomes, proteoliposomes and hybrid vesicles with comparison of catalytic rates etc. the reader is referred to previous reviews, for example^15^,^17^,^205^, and^228^ .

### 4.1 Compartmentalized Phototriggers of ATP Synthesis

Photoacid generators release protons irreversibly following photoexcitation. These proton-bearing small molecules become acidic in their excited states, releasing protons and thereby lowering the local proton concentration sometimes by several orders of magnitude. Li et al.^229^ used 8-hydroxypyrene-1,3,6-trisulfonate trisodium salt (HPTS) as a photoacid to drive photophosphorylation, Figure 11a. HPTS has a p*K*_a_ of 7.4 in its ground state which is changed to 0.5 after photoexcitation. When proton release occurred inside ATP synthase containing liposomes approximately 38 ATP molecules were produced s^–1^ ATP synthase^–1^. For that study the liposomes were templated onto hierarchical silica nanoparticles that served as both photoacid repositories and a scaffold onto which an ATP synthase containing bilayer shell was formed from electrostatic interaction with ATP synthase loaded liposomes.

**Figure 11:**
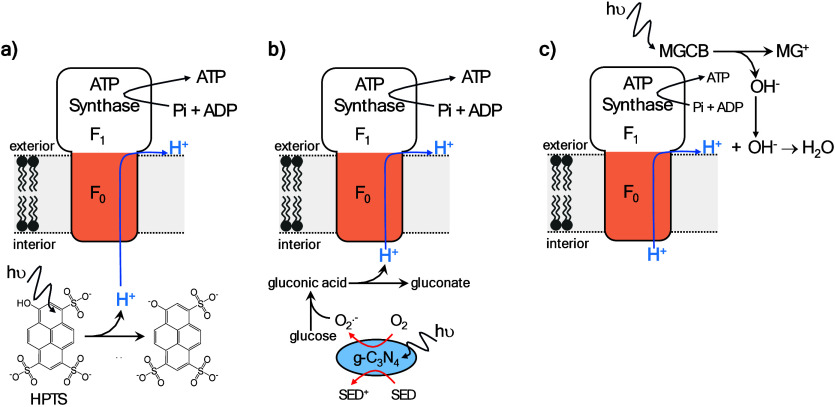
Schematic representation of ATP synthesis driven by compartmentalized phototriggers as reported by (a) Li et al.,^229^ (b) Li et al.^230^ and (c) Li et al.^235^ HPTS = 8-hydroxypyrene-1,3,6-trisulfonate trisodium salt; MGCB = malachite green carbinol base; MG^+^ = malachite green; SED = sacrificial electron donor.

Internal acidification of ATP synthase containing liposomes has also been achieved with encapsulated g-C_3_N_4_ nanoparticles as photosensitizers.^230,231^ Under irradiation, valence electrons from g-C_3_N_4_ were excited to the conduction band and holes were generated, Figure 11b. The photoenergized electrons reacted with O_2_ to produce superoxide (O_2_^·–^) which in turn converted encapsulated glucose into gluconic acid with concomitant release of protons. The ground state g-C_3_N_4_ particles were returned by oxidation of coencapsulated SEDs; polyethylenimine^230^ or the dipeptide *N*-fluorenylmethoxycarbonyl diphenylalanine.^232^ Spatial and temporal control of proton release for ATP synthesis was also demonstrated with merocyanine^233^ and 1-hydroxypyrene^234^ as photoacids. A complementary approach to *pmf* creation used the light-activated proton scavenger, malachite green carbinol base.^235^ Nanoparticles of this photobase were introduced to solutions of ATP synthase containing vesicles. Irradiation with ultraviolet light led to ATP synthesis, Figure 11c, and a pH increase of 3.5 units outside the liposomes. ATP synthesis has also been described for vesicles that use irradiated purple membranes^232^ and cross-linked PSII-bovine serum albumin microspheres^236^ to create a *pmf*.

### 4.2 Charge Transfer Conduits for ATP Synthesis and C-Fixation

As noted in Section 3, several light-driven conduits create *pmf* in response to irradiation. Incorporating these conduits in vesicles alongside ATP synthase can enable photophosphorylation. Several reviews provide excellent summaries of different vesicular approaches to photophosphorylation and the rates that have been achieved, for example^17^,^18^,^205^,^228^,^234^, and^237^ . Our Table 6 summarizes notable examples with an emphasis on biohybrid aspects.

**Table 6:** A Selection of Literature on Photophosphorylation Using Light-Activated Conduits for Charge Transfer Relevant to the Topic of This Review^a^

	Location of ATP synthesis	Comments	Rate of ATP synthesis	Ref	Year
Synthetic Conduits In Liposomes	outside	C–P–Q molecular triad with chloroplast ATP synthase into preformed liposomes.	The light saturated rate of ATP synthesis was approximately 7 ATP per second per ATP synthase.	^150^	1998
	outside	Chloroplast ATP synthase into preformed liposomes followed by addition of molecular conduit assembled around Hf-oxo clusters that inserts into the external liposome surface.	Turnover frequency for light-driven ATP synthesis 47 per min.	^151^	2020
BR in Liposomes	outside	Purple membranes and beef heart ATP synthase in liposomes prepared with soybean phospholipids.	ATP synthesis monitored through an assay that reports 594 nmol glucose-6-Pi formed per min per mg BR.	^218^	1974
	outside	Purple membranes and ATP synthase from *Bacillus* PS3 in liposomes prepared with soybean phospholipids..	ATP synthesis quantified for various assembly protocols with a maximum rate of 25 nmol ATP per min per mg ATP synthase	^238^	1977
	outside	ATP synthesis compared for liposomes prepared from *R. rubrum* ATP synthase and soybean phospholipids in combination with purple membranes or monomeric BR.	Rates of ATP synthesis with BR monomers (maximally 280 nmol ATP per min per mg ATP synthase) are 6-fold faster than for purple membranes. This was attributed to a more homogeneous distribution of BR monomers across the liposomes.	^239^	1987
	outside	Purple membranes and chloroplast ATP synthase reconstituted (1:170 molar ratio) into preformed liposomes of phosphatidylcholine and phosphatidic acid.	The maximum rate of ATP synthesis is approximately 200 nmol ATP per min per mg ATP synthase.	^240^	1992
	outside	Liposomes formed from soybean phospholipids in the presence of beef heart ATP synthase and purple membrane or monomeric BR.	A maximal rate of 58 nmol ATP per min per mg ATP synthase was obtained using monomeric BR and liposome formation by dialysis from a survey of several different assembly processes.	^241^	1995
	outside	Monomeric BR and *Bacillus* PS3 ATP synthase incorporated into liposomes comprised of phosphatidylcholine and phosphatidic acid by a variety of methods.	Including cholesterol resulted in 20-fold higher rates of ATP synthesis: 500–800 nmol ATP per min per mg ATP synthase.	^242^	1996
	outside	Monomeric BR and *Bacillus* PS3 ATP synthase were reconstituted into preformed liposomes of phosphatidylcholine, phosphatidic acid and cholesterol.	Vesicles were encapsulated in a silica-PEG sol–gel that when irradiated produced ATP at a rate of approximately 0.037 nmol ATP per min per mg ATP synthase.	^224^	2005
	outside	Monomeric BR and *Bacillus* PS3 ATP synthase reconstituted into liposomes of phosphatidylcholine and cholesterol. Enzymes were added prior to complete detergent removal and liposome formation. This resulted in 86% of BR having the working orientation (outward C-terminus) required for internal acidification during irradiation.	ATP synthesis rates were approximately 220 nmol ATP per min per mg ATP synthase.	^244^	2019
	inside	Colloidosomes composed of AuAg nanorods covered in liposomes prepared from purple membranes. Those vesicles were mixed with separately prepared liposomes containing chloroplast ATP synthase.	When irradiated for 1 h the mixture formed approximately 540 nmol ATP per mg ATP synthase inside the ATP synthase containing liposomes.	^203^	2019
	outside	Monomeric BR and recombinant *E. coli* ATP synthase into preformed liposomes of phosphatidylcholine.	ATP synthesis rates were approximately 260 nmol ATP per min per (mg ATP synthase).	^43^	2020
	outside	Purple membrane patches and *E. coli* ATP synthase incorporated into preformed phosphatidylcholine liposomes.	The maximal rate of ATP synthesis was 4500 nmol ATP per min per mg ATP synthase. This high rate was attributed to aiming for a theoretical ratio of 1 ATP synthase and 96 BR molecules per liposome with almost uniform direction. The impact of different assembly procedures on activity are reported.	^245^	2021
BR in Polymersomes and Hybrid Vesicles	outside	Vesicles prepared from a suspension containing purple membranes, *Bacillus* PS3 ATP synthase and the ABA triblock copolymer PEtOz–PDMS–PEtOz.	Approximately 1100 nmol ATP produced per mg ATP synthase over 1 h irradiation.	^44^	2005
	outside	Vesicles prepared from an ethanolic suspension containing purple membranes, *Bacillus* PS3 ATP synthase and the ABA triblock copolymer PEtOz–PDMS–PEtOz.	Irradiation for 1 h produced approximately 1150 nmol ATP inside the vesicles per mg ATP synthase. The rate of ATP formation dropped over time.	^214^	2006
	inside	Vesicles prepared from an aqueous suspension containing purple membranes, *Bacillus* PS3 ATP synthase and the ABA triblock copolymer PEtOz–PDMS–PEtOz. Irradiation for 1 h produced approximately 1300 nmol ATP inside the vesicles per mg ATP synthase.	Vesicles suspended in the water channels of a foam prepared from the detergent Tween-20 when irradiated for 1 h produced approximately 1800 nmol ATP per mg ATP synthase. In both scenarios the rate of ATP formation accelerated over time which the authors proposed was due to increased ATP binding to, and activation of, the F_1_ subunit.	^214^,^215^	2006
	inside	Monomeric BR and *Bacillus* PS3 ATP synthase in tubular vesicles prepared from an amphiphilic ABA triblock copolymer PEOXA-*b*-PDMS-*b*-PEOXA. ATP was produced inside the tubular vesicles during irradiation.	The rate of ATP production increased over time, 82 000 nmol ATP per mg of ATPase in 30 min and 27 300 nmol ATP per mg ATPase in 60 min.	^248^	2018
	outside	Rates of photophosphorylation were significantly less for monomeric BR and recombinant *E. coli* ATP synthase in polymer/polymer vesicles when compared to polymer/lipid vesicles.	ATP synthesis was 255 nmol (mg ATP synthase)^−1^ min^–1^ with 70/30 (mol %) PDMS/phosphatidyl choline and 240 nmol (mg ATP synthase)^−1^ min^–1^ for 50/50 PBd-*b*-PEG/phosphatidylcholine. However, 140 nmol (mg ATP synthase)^−1^ min^–1^, in the mixed polymer vesicles having 50/50 PDMS/PBd-*b*-PEG.	^43^	2020
PSI, PSII or RCin Liposomes	outside	PSI and ATP synthase from spinach chloroplasts in liposomes of soybean phospholipids. When irradiated with phenazine methosulfate, cyclic electron transfer supports proton transfer across the membrane.	Photophosphorylation rates approached 830 nmol ATP per min per mg chlorophyll.	^246^	1980
	outside	PSII based microspheres were formed by cross-linking to bovine serum on a CaCO_3_ template. After removal of the CaCO_3_ template, microspheres were coated with liposomes containing spinach chloroplast ATP synthase.	Irradiation for 1 h produced approximately 1100 nmol ATP per mg chlorophyll. The rate of ATP production decreased during this time.	^236^	2016
	outside	Liposomes prepared with spinach PSII, proteorhodopsin and ATP synthase from *Bacillus pseudofirmus*.	Irradiation (5 min) with white light produced 150 nmol ATP per 150 nmol chromophore.	^249^	2018
	outside	Liposomes containing RC and ATP synthase prepared from lysis of *R. sphaeroides* cells.	ATP production rate up to ∼16 μmol ATP per min per mg ATP synthase.	^250^	2021

a Abbreviations: PBd-*b*-PEG = polybutadiene_22_-*b*-poly­(ethylene oxide)_14_; PDMS = poly­(dimethylsiloxane)-*g*-poly­(ethylene oxide); PEOXA-*b*-PDMS-*b*-PEOXA = poly­(2-ethyloxazoline-*block*-dimethylsiloxane-*block*-2-ethyloxazoline); PSI = photosystem I; PSII = photosystem II; RC = reaction center.

Synthetic conduits that have been successfully used for photophosphorylation include the C–P–Q triad^149,150^ of Steinberg-Yfrach et al., Figure 6e,7c, and the Janus metal–organic layers^151^ of Hu et al., Figure 7d. Studies employing biological conduits are much more numerous. Seminal studies demonstrated photophosphorylation in liposomes that contained combinations of BR and ATP synthase, for example^218^,^238^, and^239^ . Photophosphorylation rates were found to be critically dependent on lipid composition, the ratio of BR to ATP synthase, and enzyme orientation with respect to the membrane which for BR is often heterogeneous.^212,218,224,238–245^ Photophosphorylation by PSII containing liposomes is reviewed by Jia and Li^15^ and we note that rates can be enhanced by including light-harvesting CuInS_2_/ZnS nanoparticles.^166^ When reconstituted into vesicles PSI can also drive photophosphorylation.^246,247^ When irradiated, such vesicles preformed redox cycling of a membrane permeable redox shuttle, phenazine methosulfate that carried protons across the liposome membrane, Figure 12a. The redox chemistry of the shuttle acidified the liposome interior and allowed external ATP synthesis.

**Figure 12:**
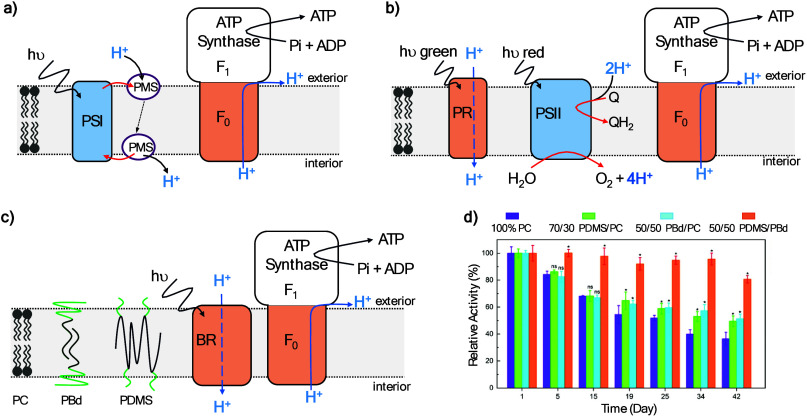
Schematic representations of ATP synthesis powered by light-driven protein conduits as reported by (a) Hauska et al.,^246^ (b) Lee et al.^249^ and (c), (d) Kleineberg et al.^43^ In b) the light primarily absorbed by PR and PSII is indicated. In c) hydrophilic (green) and hydrophobic (black) parts of the PBd-*b*-PEG and PDMS polymers are indicated. BR = bacteriorhodopsin; PBd-*b*-PEG = polybutadiene_22_-*b*-poly­(ethylene oxide)_14_; PC = phoshatidylcholine; PDMS = poly­(dimethylsiloxane)-*g*-poly­(ethylene oxide); PMS = phenazine methosulfate; PR = proteorhodopsin; PSI = photosystem I; PSII = photosystem II. Panel d) Adapted with permission from ref 43. Copyright 2020, Wiley-VCH.

Lee et al.^249^ elegantly demonstrated how rates of ATP synthesis can be controlled temporally and through the choice of irradiation wavelength. Their liposomes contained ATP synthase with two photoconverters, proteorhodopsin and plant-derived PSII, Figure 12b. Both light-harvesting proteins contributed to *pmf* formation during irradiation by white-light. The density ratios of proteorhodopsin, PSII and ATP synthase over lipid (19,000:1, 260:1, and 2,000:1, respectively) were chosen to ensure that PSII and proteorhodopsin would contribute similarly to *pmf* creation and maximize ATP synthesis. However, proteorhodopsin responds primarily to green light whereas PSII is activated by blue and red light. In addition, proteorhodopsin exhibits pH dependent bidirectional proton translocation activity. As a consequence, independent optical activation of the two photoconverters allowed dynamic control of ATP synthesis whereby red light facilitated, and green light impeded, ATP synthesis.^249^

Several studies have combined BR and ATP synthase in polymer membranes. Kleineberg et al.^43^ compared photophosphorylation rates for BR and ATP synthase in liposomes, polymer/lipid vesicles and mixed polymer vesicles. The assembly procedures aimed for 1 ATP synthase to 28 BR molecules. The polymers assembled as membranes with a thickness of approximately 5 nm and both had a hydrophilic block of poly­(ethylene oxide) (PEO). However, the hydrophobic block and architecture of the polymers differed, Figure 12c. One polymer was a diblock copolymer, polybutadiene_22_-*b*-poly­(ethylene oxide)_14_ (PBd-*b*-PEG). Another was a comb-type graft siloxane surfactant, poly­(dimethylsiloxane)-*g*-poly­(ethylene oxide) (PDMS). ATP production rates were greatest in the phosphatidylcholine liposomes, approximately 260 nmol ATP (mg ATP synthase)^−1^ min^–1^. The rates were only slightly lower in the polymer/lipid vesicles; approximately 255 nmol (mg ATP synthase)^−1^ min^–1^ with 70/30 (mol %) PDMS/phosphatidyl choline and 240 nmol (mg ATP synthase)^−1^ min^–1^ for 50/50 PBd-*b*-PEG/phosphatidylcholine. ATP production was significantly slower, 140 nmol (mg ATP synthase)^−1^ min^–1^, in the mixed polymer vesicles having 50/50 PDMS/PBd-*b*-PEG. However, protein stability was significantly enhanced in the mixed polymer vesicles compared to the other systems, Figure 12d. The rate of ATP production for the PDMS/PBd-*b*-PEG vesicles was slightly higher^43^ than that reported by Choi et al.^44^ for ATP synthase and purple membrane patches assembled in vesicles comprised of PEtOz–PDMS–PEtOz triblock copolymer, approximately 120 nmol (mg ATP synthase)^−1^ min^–1^. Dhir et al.^248^ reported ATP synthesis inside tubular vesicles comprised of the amphiphilic ABA triblock copolymers poly­(2-ethyloxazoline-*block*-dimethylsiloxaneblock-2-ethyloxazoline). Those vesicles, which contained BR and ATP synthase, had an average membrane thickness of 5 nm, external diameter around 30 nm and lengths of 50 – 450 nm. Irradiation for 1 h produced approximately 25 μmol ATP per mg ATP synthase.

Photophosphorylation has also been reported for BR containing vesicles mixed with separately prepared ATP synthase containing vesicles.^203^ In that study the operation of BR was enhanced by the presence of light-harvesting colloidosomes prepared of AuAgNPs. Protons pumped out of the BR containing vesicles created the *pmf* for ATP synthesis inside the ATP synthase containing liposomes.

Several accounts illustrate how ATP produced by photophosphorylating vesicles can be harnessed to power additional reactions.^169,170,249^ Two of those reports describe light-driven C-fixation. Lee et al.^249^ produced ATP on the external surface of liposomes containing ATP synthase, proteorhodopsin and PSII. Placed in solutions of the enzyme pyruvate carboxylase, the irradiated liposomes drove the carboxylation of pyruvate (CH_3_COCO_2_^–^) in an ATP-dependent reaction, Figure 13a. Wang et al.^169^ performed carbon fixation using a system of nested vesicles whereby photophosphorylating liposomes were encapsulated in larger liposomes, Figure 13b. Liposomes containing ATP synthase and PSII enabled external light-driven ATP production. Those liposomes, approximate diameter 0.2 μm, were encapsulated in giant liposomes with approximate diameter 16 μm. Also encapsulated in the giant liposomes were phycocyanin, to enhance the light-harvesting properties of PSII and three enzymes that enabled C-fixation through an ATP-dependent cascade reaction. The carbon fixation efficiency was 74% for 1 h irradiation. The formation of acetyl-CoA as a product of this reaction is also of note. Since this molecule is a cosubstrate for many enzymes there are opportunities to deliver additional enzyme catalyzed reactions and a greater product range.

**Figure 13:**
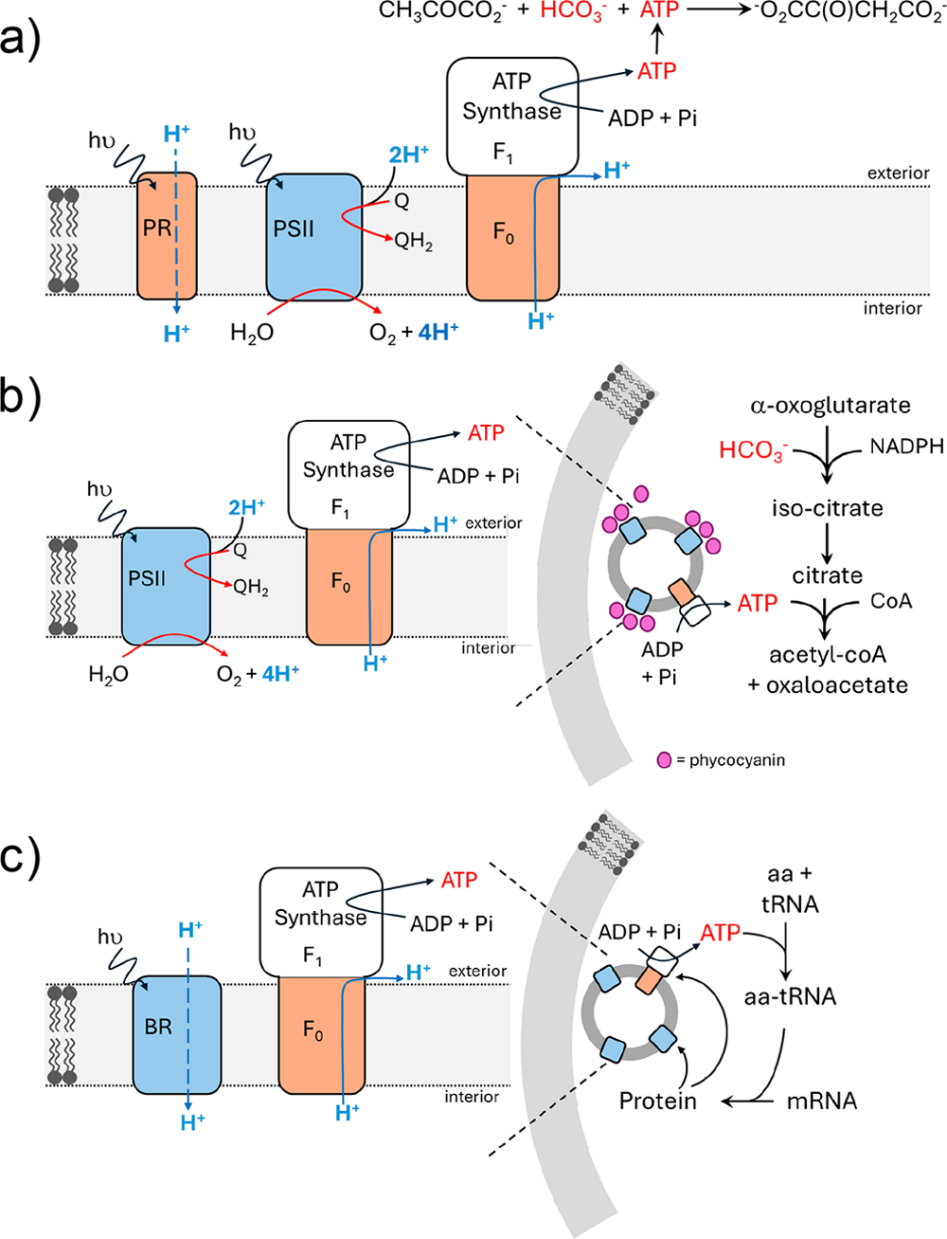
Schematic representations of photoactive liposomes that harness photophosphorylation for downstream chemistry: carbon-fixation reported by (a) Lee et al.^249^ and (b) Wang et al.^169^ and protein synthesis (c) reported by Berhanu et al.^244^ aa = amino acid; BR = bacteriorhodopsin; PSII = photosystem II; tRNA = transfer RNA; mRNA = messenger RNA.

In a related approach to C-capture, Gao et al.^170^ employed thylakoids, with light-harvesting properties enhanced by CdTe quantum dots, for the light-dependent production of NADPH, NADH and ATP. This enabled conversion of CO_2_ to formate and methanol when the photoactive units were encapsulated with appropriate enzymes in water-in-oil microdroplets stabilized by block copolymer surfactant. Additional routes to C-fixation in synthetic systems are presented and/or reviewed by the following authors Cai et al.,^251^ Miller et al.,^252^ Park et al.,^237^ Schwander et al.,^253^ Sen Thepa et al.,^254^ Shi et al.^255^ and Wendell et al.^256^

In an elegant step toward self-replication and repair of photoactive liposomes, Berhanu et al.^244^ reported light-driven production of BR and ATP synthase within nested vesicles, Figure 13c. Millimolar amounts of ATP were produced inside giant vesicles with an approximate diameter of 10 μm by entrapped liposomes of approximately 0.2 μm diameter that contained BR and ATP synthase. The highest ATP photosynthesis was recorded with 176 μM BR and 1 μM ATP synthase. The photosynthesized ATP was subsequently used to produce BR and the F_0_ component of ATP synthase. The newly synthesized BR and F_0_ protein were localized to the internal liposome membranes and increased photophosphorylation through a positive feedback loop.

Vesicle dependent photophosphorylation machineries driving ATP-dependent DNA transcription,^250^ actin polymerization^248,249^ and motility^245^ have also been reported. Taken together, these final examples have showcased vesicular systems employing photoactive vesicles to power different dark-reactions. Some of this chemistry is achieved with a single enzyme. Sometimes the desired chemistry is achieved through a series of reactions performed by including several different enzymes. Several of the examples employ nested multiwalled vesicles, i.e., vesicles within vesicles, to fully exploit the advantages of spatially defined reaction compartments. Together these reports serve to illustrate the opportunities for vesicular approaches to solar-to-chemicals conversion that can be developed in advancing biohybrid approaches.

## 5 Summary and Outlook

Since initial studies on ion diffusion and light-induced phenomena across lipid membranes in the 60s,^54,75,85,90,91^ our understanding of natural photosynthesis and ability to mimic its chemistry in photocatalytic biohybrid vesicles has steadily progressed. Early studies used biological small molecules such as chlorophylls and natural quinones, leading to increased understanding and then utilization of more complex systems, including transmembrane protein complexes and synthetic photosynthetic ‘wires’ that transverse the membrane. Subsequent developments combined antennae, photosensitizers, (bio)­catalysts and transmembrane charge transporters (mediators and conduits), sometimes with membranes containing synthetic amphiphiles, to create a greater variety of photocatalytic systems, with the key principle being the capture of light energy to drive transmembrane charge transfer.

With photocatalytic biohybrid vesicles becoming increasingly complex, continued progress will require an ever-higher level of integration across disciplines. The purification and reconstitution of membrane-protein complexes requires, more often than not, special attention. This Review, for example, cited examples where minor changes in the reconstitution protocol changed the orientation of rhodopsins and thereby the direction of proton pumping.^213,214,216,217,223^ There is much opportunity to interface synthetic membrane soluble charge carriers with catalysts performing a wider range of chemical conversions. We postulate that the current fast development in the field of polymersomes, lipid–polymer hybrid vesicles and other vesicle structures, as well as the synthesis of light-harvesting compounds and photosensitizers will lead to important progress in photocatalytic biohybrid vesicles.

Several important future milestones can be identified for the field of photocatalytic biohybrid vesicles, both related to their sustainability and applicability. For redox catalysis, a significant milestone is to achieve valuable chemistry in both half-reactions. Most systems reported to date only focus on one-half-reaction, requiring either a sacrificial electron donor or acceptor to complete the redox cycle. Cataloguing the true benefits of biohybrid vesicles requires us to phase out these sacrificial compounds. Continued development in the (bio)­catalysis of oxidoreduction reactions thus remains important. Another important consideration is the membrane permeability of some of the potential products of photocatalysis, such as hydrogen or oxygen. Biohybrid vesicles might not be able to compartmentalize the two half-reactions and prevent back reactions if vesicles are permeable to the products.

A milestone for all photocatalytic biohybrid vesicles is their easy, reproducible manufacture. For many photocatalytic biohybrid systems, the manufacture is labor intensive, while results are sometimes not easily transferable between academic laboratories, let alone transferable to the biotechnological industry. Technological advances in the field of artificial or minimal cells will be paramount to solve this bottleneck. Improvements in the self- or directed-assembly of biohybrid vesicles, preferably using commercial devices or products, will improve transferability between laboratories and disciplines. Continued progress in microfluidics and other droplet-based vesicle generation techniques,^257–261^ as well as the (commercial) availability of (microfluidic) devices, could prove important for the production of homogeneously sized vesicles in which the lumen and extravesicular content are reproducibly controlled. Other methods for formation of complex vesicles, such as genetically engineered exosomes, extracellular vesicles or outer membrane vesicles shedded by cells, could be explored.^262,263^

A further milestone is the improvement in yield, both in terms of quantum yield, turnover frequencies and turnover numbers. Many studies, however, do not report such quantitative assessments, making direct comparison impossible. Several works also do not provide sufficient detail on experimental setups, with parameters such as light source and light intensity not or insufficiently described. For the field to progress, we recommend that the evaluation of quantum yield, stability and turnover numbers are provided routinely and in a consistent manner to enable quantitative comparison. Indeed, a timely proposal for standardized reporting of data in light-driven catalysis was recently published to provide a framework for such comparisons.^264^

From this Review it is clear that the current-state-of-the-art in photocatalytic vesicle research lies in academic or curiosity-driven research, with a large focus on understanding the principles of natural photosynthesis by mimicking its (photo)­chemistry. In Section 4.2, early and emerging applications of biohybrid vesicles as a light-driven energy source in synthetic or artificial cells are presented. For instance, a property of photocatalytic vesicles that has generated significant success has been the storage of light-energy in the form of electrochemical *pmf* gradients. This has been particularly explored for the generation of ATP by ATP synthase. ATP is a useful biological fuel for powering downstream biochemical conversions. An intriguing vision that is beginning to be explored is photo-ATP generation by biohybrid vesicles inside larger artificial cells.^169,170,249^ Here, the biohybrid vesicles perform energy conversion in a manner analogous to an artificial chloroplast or mitochondria. This approach should ultimately be able to power protein and metabolite synthesis, the self-replication of artificial cells and C-capture for chemicals synthesis. We expect that the performance of such systems will be enhanced by integration with synthetic materials in photocatalytic biohybrid vesicles. Still, many bottlenecks need to be solved if expertise in photocatalytic vesicles is to be transferred to delivering biotechnological or biomedical applications. In spite of these challenges, photocatalytic biohybrid vesicles hold much promise, for instance, in added-value chemical (bio)­synthesis, solar fuel production and light-driven, targeted drug release. Each of these potential applications faces different challenges. For solar-fuel production, we foresee an immense challenge in the scalable production of robust biohybrid vesicles with long operation times under illumination conditions. For biomedical applications, reproducible manufacturing procedures will need to be developed, while potential toxicity issues of vesicle components will need to be determined, and addressed. In our view, therefore, the most promising application of photocatalytic biohybrid vesicles lies in added-value chemical synthesis. Here, the challenge lies in the development of added functionality over those capabilities already provided by biocatalysts ‘free’ in the reaction medium.

## Abbreviations

A_ox_: acceptor of electrons
AA: ascorbic acid
ADP: adenosine 5′-diphosphate
ATP: adenosine 5′-triphosphate
BLM: black lipid membrane
bpy: 2–2’-bipyridine
BR: bacteriorhodopsin
BQ: benzoquinone
BV: 1–4-bis­(1,2,6-triphenyl-4-pyridyl)­benzene (benzyl viologen)
C_*n*_V: alkyl viologen
Chl *a*: chlorophyll *a*
CO_2_: carbon dioxide
C–P–Q: carotenoid polyene-tetraarylporphyrin-napthoquinone
Cyt *b*_6_ *f*: cytochrome *b*_6_ *f*
Cyt c: cytochrome *c*
D_red_: donor of electrons
DBMIB: 2–5-dibromo-3-methyl-6-isopropylbenzoquinone
DT: dithionite
DTT: dithiothreitol
EDTA: ethylenediaminetetraacetic acid
EPR: electron paramagnetic resonance
H_2_ase: hydrogenase
Fc: ferrocene
Fd: ferredoxin
FDH: fructose dehydrogenase
FMN: flavin mononucleotide
FNR: ferredoxin-NADP^+^ oxidoreductase
g-N-CDs: graphitic nitrogen-doped carbon dots
H_2_ase: hydrogenase
HER: hydrogen evolution reaction
HPTS: 8-hydroxypyrene-1,3,6-trisulfonate trisodium salt
HQNO: 2-heptyl-4-hydroxyquinoline N-oxide
iCHELLs: inorganic chemical cells
ITS: indigotetrasulfonic acid
LHCs: light-harvesting complexes
MitoQ: Triphenylphosphonium ubi- and plastoquinones
MG: malachite green
MGCB: malachite green carbinol base
MK: menaquinone
MMP^+^: 1-methoxy-*N*-methylphenazinium
mRNA: messenger RNA
MtrAB: outer membrane spanning porin-cytochrome complex of *S. oneidensis*
MtrC: extracellular cytochrome of *S. oneidensis*
MtrCAB: outer membrane complex of *S. oneidensis* comprised of MtrC and MtrAB
MV: methyl viologen
NAD^+^: nicotinamide adenine dinucleotide
NADH: dihydronicotinamide adenine dinucleotide
NADP^+^: nicotinamide adenine dinucleotide phosphate
NADPH: dihydronicotinamide adenine dinucleotide phosphate
NR: nanorod
NP: nanoparticle
O-PDI: oligo­(p-phenylene)–N–N-perylenediimide
PBd-*b*-PEG: polybutadiene_22_-*b*-poly­(ethylene oxide)­14
PCu: plastocyanin
PDMS: poly­(dimethylsiloxane)-*g*-poly­(ethylene oxide)
PEG: poly­(ethylene glycol)
PEtOz–PDMS–PEtOz: [poly­(2-ethyl-2-oxazoline)-*b*-poly­(dimethylsiloxane)-*b*-poly­(2-ethyl-2-oxazoline)]
PEO: poly­(ethyl oxide)
PEOXA-*b*-PDMS-*b*-PEOXA: poly­(2-ethyloxazoline-*block*-dimethylsiloxane-*block*-2-ethyloxazoline)
Pi: phosphate
*pmf*: proton motive force
PMS: phenazine methosulfate
POM: polyoxometalate
Pp *a*: pheophytin *a*
PR: proteorhodopsin
PS: photosensitizer
PSI: photosystem I
PSII: photosystem II
PQ: plastoquinone
PQH_2_: plastoquinol
Q: quinone
RC: reaction center
SED: sacrificial electron donor
TCNQ: 7,7,8,8-tetracyanoquinodimethane
TEOA: triethanolamine
TMBQ: trimethylbenzoquinone
TMPD: N,N,N’,N’-tetramethyl-p-phenylenediamine
TMPyP: 5,10,15,20-tetra­(4-*N*-methylpyridyl)-porphyrin
TPP: 5,10,15,20-tetraphenyl-porphyrin
TPPS: 5,10,15,20-tetra­(4-sulfonatophenyl)-porphyrin
tRNA: transfer RNA
UQ: ubiquinone
WST: 2-(4-iodophenyl)-3-(4-nitrophenyl)-5-(2,4-disulfophenyl)-2H-tetrazolium anion
XTT: 2,3-bis­(2-methoxy-4-nitro-5-sulfophenyl)-2*H*-tetrazolium-5-carboxanilide
ΔΨ: transmembrane potential
ΔpH: pH gradient
